# Scaling-Up an Aqueous Self-Degassing Electrochemically Mediated ATRP in Dispersion for the Preparation of Cellulose–Polymer Composites and Films

**DOI:** 10.3390/polym14224981

**Published:** 2022-11-17

**Authors:** Francesco De Bon, Inês M. Azevedo, Diana C. M. Ribeiro, Rafael C. Rebelo, Jorge F. J. Coelho, Arménio C. Serra

**Affiliations:** Department of Chemical Engineering, Centre for Mechanical Engineering, Materials and Processes, University of Coimbra, Rua Sílvio Lima-Pólo II, 3030-790 Coimbra, Portugal

**Keywords:** *e*ATRP, self-degassing, polyacrylamides, cellulose, scale-up

## Abstract

Electrochemically mediated atom transfer radical polymerization (*e*ATRP) is developed in dispersion conditions to assist the preparation of cellulose-based films. Self-degassing conditions are achieved by the addition of sodium pyruvate (SP) as a ROS scavenger, while an aluminum counter electrode provides a simplified and more cost-effective electrochemical setup. Different polyacrylamides were grown on a model cellulose substrate which was previously esterified with 2-bromoisobutyrate (-BriB), serving as initiator groups. Small-scale polymerizations (15 mL) provided optimized conditions to pursue the scale-up up to 1000 mL (scale-up factor ~67). Cellulose-poly(*N*-isopropylacrylamide) was then chosen to prepare the tunable, thermoresponsive, solvent-free, and flexible films through a dissolution/regeneration method. The produced films were characterized by Fourier-transform infrared (FTIR), scanning electron microscopy (SEM), dynamic scanning calorimetry (DSC), and thermogravimetric analysis (TGA).

## 1. Introduction

**Scheme 1:**
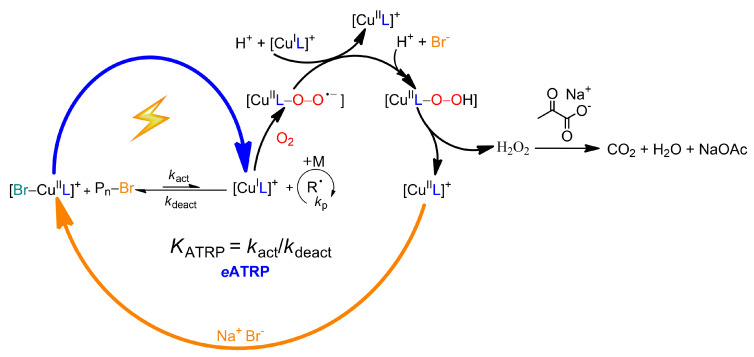
Proposed mechanism of *e*ATRP with embedded O_2_ scavenging cycle for self-degassing in H_2_O. M is the monomer, while *k*_act_, *k*_deact_, and *k*_p_ are the activation, deactivation, and propagation rate constants, respectively. Termination reactions from R^.^ are omitted for clarity.

Cellulose is the most abundant biopolymer on Earth [1,2,3]. It consists of linear β-1,4 D-glucose units, rich with hydroxyl (–OH) active groups that can form inter- and intramolecular bonds between cellulose chains, causing strong hydrogen-bond networks [2]. Thanks to these intramolecular bonds, cellulose chains are relatively stable and exhibit high axial stiffness. Throughout human history, cellulose has been used extensively in the manufacturing of printing paper, packaging, textiles, health products, and pharmaceuticals. Cellulosic materials are therefore of economic importance due to their chemical uniqueness, shape flexibility, mechanical strength, and biodegradability [4]. As a well-known natural biopolymer, it has numerous advantages such as low cost, renewability, relatively easy processing, and biodegradability. Mechanical performances as well as dielectricity, piezoelectricity, and convertibility are also highly appreciated properties [3]. Due to all these properties, cellulose is widely used as a substrate for numerous applications. The richness of –OH groups has been explored to functionalize cellulose with polymerization initiation sites that can be utilized by the most commonly used reversible deactivation radical polymerizations (RDRP), especially atom transfer radical polymerization (ATRP) [5,6,7]. However, most reactions are limited to laboratory scale and academic settings. The compelling and ever-growing need for greener applications is one of the recognized ways to make ATRP a tool of ecological transition [8]. The need for sustainable chemistry, green materials, and improved chemical synthesis led us to focus on a way to scale up ATRP using cellulose as a starting substrate, providing reactors and reactions as close as possible to their industrial implementation [6,9]. ATRP gives the opportunity to design well-defined and often complex polymer structures. ATRP is a catalytic process usually mediated by Cu complexes and is tolerant to a variety of functional groups, solvents [9], and more recently oxygen [10,11,12,13,14]. Active copper catalysts with strong negative reduction potential allowed for well-controlled polymerizations at catalyst loadings as low as 10–100 parts per million (ppm), based on monomer concentration, in H_2_O and organic solvents. Among all externally controlled ATRP methods [15], electrochemically mediated ATRP (*e*ATRP), introduced in 2011 [16], bloomed in the last years [17]. The *e*ATRP mechanism relies on a tight control of the redox process occurring at the electrode surface by specifying an applied potential (*E*_app_), current (*I*_app_), or total charge (Q) transmitted. These parameters can be set a priori to constrain the equilibrium and allow tight control of polymerization. *e*ATRP is also a redox-switchable process, as it can be stopped and (re)initiated (ON/OFF toggle) by modulating the electrochemical stimulus or even by turning off the cell, allowing temporal control of the process [18,19]. *e*ATRP provided excellent results in the synthesis of well-defined architectures, which are becoming increasingly attractive. Although some limitations remain, particularly due to its elaborate structure, *e*ATRP has become increasingly user-friendly. The scale-up of *e*ATRP depends strongly on oxygen tolerance (Scheme 1). Indeed, [Cu^I^L]^+^ reacts with dissolved molecular O_2_ to form reactive oxygen species (ROS) such as peroxides and hydroperoxides, eventually releasing hydrogen peroxide. H_2_O_2_ is a poison because it decomposes to produce hydroxyl radicals (-OH), which are among the most powerful oxidants known [20]. Due to their extreme reactivity, it reacts unselectively and rapidly with almost all chemicals in the environment. Therefore, a ROS scavenging agent is added to the reaction medium to neutralize any ROS or H_2_O_2_. Sodium pyruvate (SP) is probably the most commonly used hydrophilic ROS scavenger. Methyl benzoylformate, on the other hand, is a lipophilic ROS scavenger suitable for organic solvents [21].

**Scheme 2:**
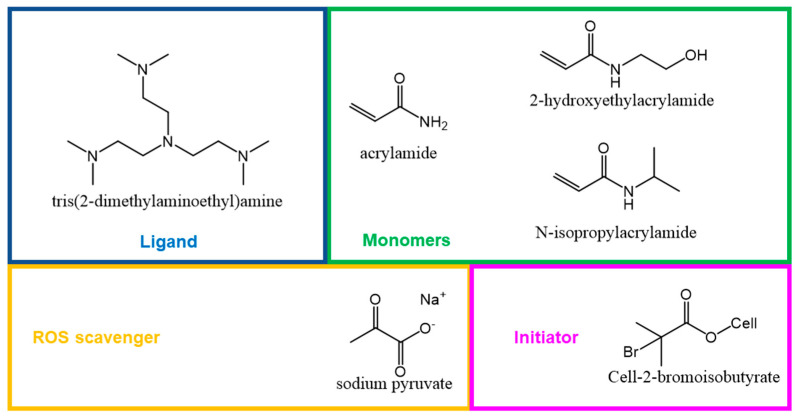
Ligands, monomers, ROS scavenger, and initiator used in this work.

Thanks to oxygen tolerance, we reported in earlier works the scale-up of simplified electrochemically mediated ATRP (*se*ATRP) up to 15 L [10] and of photoinduced ICAR ATRP up to 2 L [11]. *se*ATRP is an electrochemically simplified version of *e*ATRP: the difference between classical *e*ATRP and simplified *e*ATRP is the use of a sacrificial Al anode instead of an inert Pt anode, which instead requires a separate electrode compartment to avoid the reoxidation of electrogenerated [Cu^I^L]^+^. The use of such an anode eliminates the need for a separate electrode compartment, a prerequisite necessary for economic feasibility of the scale-up and to decrease the ohmic drop. The solubilization of cellulose has been extensively studied [22,23]. There are solvents, such as aqueous sodium hydroxide/urea [24], *N*-methylmorpholine-*N*-oxide (NMMO), *N,N*-dimethylacetamide/lithium chloride (DMAc/LiCl) mixtures, ionic liquids (ILs) [25], and deep eutectic solvents (DES) [26] that can effectively break the hydrogen bonding network and dissolve cellulose. Nevertheless, most of them are not compatible with an ATRP scale-up. For example, NMMO dissolves cellulose and serves as a solvent, but it is also an oxidizing agent and would oxidize all [Cu^I^L]^+^ to [Cu^II^L]^+^. *N,N*-dimethylacetamide/lithium chloride (DMAc/LiCl) mixtures contain such a high concentration of Cl^−^ anions that they significantly interfere with ATRP (chloride anions act simultaneously on activation and deactivation). Many ionic liquids are also incompatible with ATRP because they either contain a vinyl group (such as 1-allyl-3-alkylimidazolium ionic liquids) that can react with and quench radicals, or they contain chlorides or other counter-anions that are incompatible with ATRP (such as dicyanamides) [27]. In addition, the solubility of cellulose in such ionic liquids is usually low or very low and requires high temperatures (high enough to boil away typical monomers such as methyl (meth)acrylate or butyl acrylate) [27]. Finally, none of the above solvents is economically affordable on a large scale. For these reasons, the scale-up of an aqueous *se*ATRP under dispersion conditions should be considered. Dispersion has several advantages, but the most important is the ease of recovery of the material after polymerization. Another goal of this work is that once the material is synthesized, it can be easily used for other purposes. For the preparation of films, the class of conjugated acid-base ionic liquids (ABILs) has been developed, which has expanded the range of solvents for the preparation of cellulosic materials due to their chemical and thermal stability, low volatility, high solvation capacity, and potential recyclability. We have recently reported a route for the formation of cellulose films using cellulose dissolved in the ABIL tetramethylguanidinium acetate [TMG][OAc] [28]. This work proceeds in two phases: (1) the synthesis of suitable cellulose-based materials by a self-degassing, large-volume, galvanostatic *se*ATRP up to 1000 mL, and (2) the use of some synthesized materials to tune the properties of cellulose-based films using a dissolution/regeneration method. The ligand, *N*-alkylacrylamides, the ROS scavenger, and a schematic representation of the model cellulosic substrate used in this work are shown in Scheme 2:

Our work shows that large-scale, self-degassing, *se*ATRP can be used to prepare relevant amounts of cellulose-based materials to be used in the preparation of cellulose films. Resultant films are then characterized chemically, physically, and thermally.

## 2. Materials and Methods

### 2.1. Materials

Copper(II) bromide (CuBr_2_; Acros Organics, Geel, Belgium, 99.999%), potassium hydrogen phosphate (Honeywell Fluka, Geel, Belgium, ≥98%), anhydrous sodium dihydrogen phosphate (Honeywell Fluka, Geel, Belgium, ≥98%), tris(2-aminoethyl)amine (TREN, Acros Organics, Geel, Belgium, 96%), pyridine 2-carboxaldehyde (Merck, Schnelldorf, Germany, 98%), bis(2-picolyl)amine (Merck, Schnelldorf, Germany 98%), sodium triacetoxyborohydride (TCI Chemicals, Zwijndrecht, Belgium, 98%), 2-bromoisobutyryl bromide (BriB, TCI Chemicals, Zwijndrecht, Belgium, 98%), sodium bromide (NaBr, Sigma Aldrich, Schnelldorf, Germany, 99%), acrylamide (AAm, Acros Organics, Geel, Belgium, extra pure 98.5+%), aqueous formaldehyde (Panreac, Castellar del Valès, Spain, 37 vol%), acetonitrile (Sigma Aldrich, Schnelldorf, Germany, HPLC grade, 99.9%), sodium hydroxide (JMGS, Odivelas, Portugal, 99%), pyruvic acid (TCI Chemicals, Zwijndrecht, Belgium, 99%), sodium borohydride (Alfa Aesar, Kandel, Germany, 98%), hexane (JMGS, Odivelas, Portugal, 99.9%), and chloroform (JMGS, Odivelas, Portugal, 99.9%) were used as received. Avicel^®^ PH-101 (Sigma Aldrich, Schnelldorf, Germany, ~50 µm particle size, average DP = 240, average *M*_n_ = 38.9 kg/mol) was boiled in 1 M NaOH for 3 h, washed with water, acetone, and finally dried. *N*-isopropylacrylamide (NIPAAm, TCI Chemicals, Zwijndrecht, Belgium, 99%) was recrystallized twice from hexane to remove the inhibitor. *N*-hydroxyethyl acrylamide (HEAAm, TCI Chemicals, Zwijndrecht, Belgium, 98%) was passed through a column filled with basic alumina to remove the inhibitor and stored at −18 °C in an amber bottle. Deionized water was obtained by reverse osmosis. Aluminum metal wire (Alfa Aesar, Kandel, Germany, 99.9%), used as a counter electrode, was wrapped to obtain a coil to be immersed into the polymerization mixture. The 2.0 L reactor was purchased from BacoENG (, Hollywood, FL, USA), while the compact 50 mL reactor was fabricated at the Department of Chemical Sciences of the University of Padova, Italy. The cryostat was a Witeg WCR-12 circulator loaded with a water/ethylene glycol bath. 

### 2.2. Methods

**Instrumentation.** Small volume (15 mL) potentiostatic electrolyses were performed into a five-neck hearth-shaped glass cell (Pine Research, Durham, NC, USA), equipped with three electrodes, and connected to a BioLogic SP150 potentiostat/galvanostat, and computer with EC-Lab software (BioLogic, Sevssinet-Pariset, France). Cyclic voltammetry (CV) experiments were performed on a glassy carbon (GC) disk electrode (Pine Research), together with a Pt wire counter electrode (CE, Pine Research), an Ag/AgCl (3 M) reference electrode, or a saturated calomel electrode (SCE). Before each experiment, the GC disk was cleaned by polishing with a 0.25-µm alumina abrasive paste, followed by a 5 min ultrasonic rinse in ethanol. For 15 mL electrolysis, the working electrode (WE) was a Pt mesh (Alfa Aesar, 99.9% metal basis) with an estimated geometric area of ~6 cm^2^. The mesh was cleaned by sonication in concentrated HNO_3_ (15 min) before each experiment and rinsed with abundant water and acetone. The CE was a 14 cm aluminum wire (Alfa Aesar, 99% metal basis) immersed directly in the polymerization mixture. The cell was thermostated at the desired reaction temperature (*T* = 0–10 °C) using a cooling circulating liquid. Unless otherwise stated, all experiments were performed after only the headspace was vented with N_2_. Two SS304 reactors were used for the industrial-like polymerizations: compact 50 mL and 2.0 L. The compact reactor was equipped with an Al rod CE, a pressure gauge, a pipe for optional vacuum/gas cycling, and a sampling point. The 2.0 L reactor was equipped with a 500 cm Al coil that served as an anode, a sampling point, a pressure gauge, and a tube pipe with an open/close device for optional vacuum/gas cycles. Stirring was provided magnetically by PTFE-coated rare earth magnets of octahedral form for the 50 mL compact reactor and cross-shaped for the 2.0 L reactor. Additional details and pictures of the reactors are given in the Supporting Information (Section S6). For the water-soluble polymers (PAAm and PHEAAm), the number average molecular weight (*M*_n_^GPC^) and *Ɖ* = *M*_w_/*M*_n_ values were determined by gel permeation chromatography (GPC), by using a Malvern OmniSEC Resolve/Reveal GPC, equipped with a refractive index detector and Agilent Aquagel-OH 30 and 50 columns (300 mm × 8 µm) connected in series, protected by an Agilent Aquagel-OH guard column (8 µm). The column compartment and the detector were thermostated at *T* = 35 °C. The eluent was a 0.02 M phosphate buffer + 0.02 wt% NaN_3_ (pH = 7.4) at a flow rate of 1 mL/min. The column system was calibrated with six narrow poly(sodium methacrylate) (PNaMA) standards (*M*_n_ = 1310–160,000 Da). The molecular weight parameters of PNIPAAm were determined a gel permeation chromatography setup from Viscotek (Viscotek TDAmax, Houston, TX, USA) equipped with a differential viscometer (DV) and right-angle laser-light scattering (RALLS, Viscotek) and refractive index (RI) detectors. The column set consisted of a PLgel 5 µm guard column followed by one Viscotek T5000 column and one Viscotek T4000 column. A dual piston pump was set with a flow rate of 1 mL/min. The eluent (DMF + 0.03% LiBr) was previously filtered through a 0.2 µm filter. The analysis was carried out at 60 °C using an Elder CH-150 heater. Prior to injection (100 µL), the samples were filtered through a 0.2 µm pore size PTFE membrane. The system was calibrated with six narrow poly(methyl methacrylate) standards (*M*_n_ = 4520–50,350 Da). Molecular weight (*M*_n_^GPC^) and *Đ* of the synthesized polymers were determined by multidetector calibration (*dn/dc*_PNIPAAm_ = 0.087) using the OmniSEC software version: 4.6.1.354.

Monomer conversion was determined by ^1^H-NMR spectroscopy with a Bruker Avance III HD 400 MHz instrument, using D_2_O as solvent and 2 vol% DMF as the internal standard. 

To analyze films surface morphology, samples were examined by scanning electron microscopy (SEM). The surfaces were coated with gold and analyzed in a field emission scanning electron microscope (FESEM), ZEISS MERLIN Compact/VPCompact, Gemini II. Thermal stability of films was studied by thermogravimetric analysis (TGA) that was carried out using NETZSCH STA 44F5 (Netzsch, Selb, Germany). Samples were heated in a temperature range of 30 °C to 500 °C at a heating rate of 10 °C/min under nitrogen purge flow. Additionally, thermal behavior was evaluated by differential scanning calorimetry (DSC) performed in a NETZSCH DSC 204 F1 Phoenix model (Netzsch, Selb, Germany). All samples were analyzed in an aluminum pan with an ordinary closed aluminum lid, in a dry nitrogen environment with a purge flow and a heating/cooling rate of 10 °C·min^−1^. The samples were cooled from room temperature to −60 °C, followed a heating cycle to 250 °C. Then, the samples were cooled again to −50 °C and heated up again to 200 °C. All the values were collected from second heat flux curve.

Contact angle was determined by the sessile drop technique, using an optical tensiometer Theta Flex (Biolin Scientific, Manchester, UK) with an image resolution of 1984 × 1264 pixels, 3009 fps maximum measuring speed, and ±0.1° accuracy. The water drop volume was 4.0 μL, and measurements were performed in a controlled temperature ambient (25 °C). Before testing, films were placed into an oven at 25 or 50 °C. The equipment allows for monitoring the spreading contact angles until their final state. Then, the drop profile was analyzed following the Young–Laplace equation and all angles were calculated.

### 2.3. Methods

**Synthetic procedures**. Tris(2-dimethylaminoethyl)amine (Me_6_TREN) was prepared by reductive methylation of TREN according to a published procedure [29]. Tris(2-pyridylmethyl)amine (TPMA) was prepared by sequential amination reduction of pyridine 2-carboxaldehyde and 2-picolylamine with sodium triacetoxyborohydride, according to a published procedure [30]. Sodium pyruvate was prepared from pyruvic acid and sodium hydroxide according to a published procedure [31].

Synthesis procedures for cellulose-BriB. 

Cellulose-BriB macroalkyl halide initiator was prepared in a two-step procedure. Avicel^®^ PH-101 cellulose was chosen as model cellulose substrate for the preparation of the cellulose derivatives. This microcrystalline cellulose (MCC) is a purified and partially depolymerized α-cellulose produced by acid hydrolysis of high-quality pulp. This further purified MCC was then reacted with 2-bromoisobutyryl bromide (2-BriB) in the presence of triethylamine (TEA) as a base in ethyl acetate to introduce bromoisobutyrate (-BriB) groups to allow polymer growth by *se*ATRP. The two steps are the following:

*1.*  *First step.* Avicel (40 g) was introduced into a 2 L borosilicate beaker equipped with a magnetic PTFE stir bar. To Avicel was added 1 L of deionized water and 40 g of NaOH. The contents of the flask were stirred, heated to boiling, and kept boiling for at least three hours. **This hot solution of NaOH is corrosive and dangerous for skin and the eyes. Operations should be done only inside a well-ventilated fume hood and only by very well-trained personnel.** During the boiling, a dark brown liquor of water-soluble impurities formed. After boiling and cooling to room temperature, cellulose was recovered by filtration and washed with water until the pH of the washing became neutral. Cellulose was then washed one more time with acetone, and dried inside the fume hood at room temperature. Yield = 94%.
*2.*  *Second step*. To prepare cellulose-BriB of different types, 2-bromoisobutyryl bromide (BriB) was added in two different amounts (H and L, respectively, see Table 1). The pretreated Avicel was transferred inside a 500 mL three necks round flask with a PTFE stir bar and suspended in ethyl acetate. The flask was put under nitrogen flow for 30 min and stirred. Then, triethylamine was added to the suspension by syringe. After that, 2-bromoisobutyryl bromide was added to the suspension dropwise under high stirring by syringe. A white precipitate of triethylamine salts, distinguishable from cellulose, started to appear, indicating the reaction of cellulose hydroxyls with 2-BriB. The suspension also warmed up and thickened. After the addition was completed, the suspension was let to react at room temperature overnight. The day after, the suspension was filtered to recover cellulose, washed with acetone, then water, and then again acetone. The filter cake was transferred first to dry inside the fume hood and then into a vacuum oven at 40 °C.

Typical procedure of self-degassing *se*ATRP of Aam at 15 mL scale on cellulose-BriB: The electrochemical cell was loaded with 12.5 mL of aqueous phosphate buffer (0.012 M phosphates adjusted to pH = 7.4), 1 mL of 15 mM aqueous stock solution of CuBr_2_ (15 μmol), 16.0 μL of Me_6_TREN (60 μmol), 1.5 g of Aam (211.03 mmol), 153.75 mg of NaBr (1.5 mmol), 82.53 mg of sodium pyruvate (0.75 mmol), and 0.3 mL of DMF used as an internal standard for NMR analysis. The cell was thermostated at *T* = 0 °C and then the headspace was degassed with N_2_ for 10 min. The polymerization mixture was **not** degassed. A CV of the catalyst was recorded to measure its reduction potential. Then, the selected cellulose macroalkyl halide initiator was added (1.5 g) and again a CV was recorded for the catalytic system. Polymerization was then started by applying the selected *E*_app_. Samples were taken at regular intervals using non-degassed syringes to measure monomer conversion. The final average number molecular weight and dispersity of PAAm-Br was obtained after cleaving from cellulose the polymer by hydrolysis of the BriB ester bond with 2 wt% KOH.

Procedure of self-degassing *se*ATRP of AAm at 500 mL scale on cellulose-BriB: The electrochemical reactor was loaded with 475 mL of deionized water, Na_2_HPO_4_ (890.1 mg, 5 mmol), and KH_2_PO_4_ (122.5 mg, 0.9 mmol) to obtain a 0.012 M phosphate buffer and was adjusted to pH = 7.4. Then, 55.84 mg of CuBr_2_ (0.25 mmol), 0.234 mg of Me_6_TREN (1 mmol), 50 g of AAm (0.703 mol), 5.124 g of NaBr (0.5 mol), 2.75 g of sodium pyruvate (0.25 mol), and 7.5 g of the cellulose substrate. 10 mL of DMF was added as the NMR internal standard. Stirring was kept at 150 rpm to homogenize the mixture during additions. The reactor was thermostated at *T* = 10 °C and then the headspace was vented with N_2_ for 5 min by opening the purge. After that, the vent was closed, the reactor sealed, and the stirring gradually increased to 250 rpm. The polymerization was started by applying the required electrolysis program (see Table 4 and Section S1). The reaction was monitored by taking samples periodically at appropriate time intervals using non-degassed syringes to measure the monomer conversion. The final average number molecular weight and dispersity of PAAm-Br was determined by cleaving from cellulose the polymer by hydrolysis of the BriB ester bond with 2 wt% KOH.

Precipitation and isolation of the materials: After the polymerization, cellulose-*g*-PAAm was isolated by precipitation in acetone and filtrated. Cellulose-*g*-PNIPAAm was removed from the mixture by heating at 50 °C. The material turned insoluble and was separated by filtration. This process was repeated twice. Cellulose-*g*-PNIPAAm and cellulose-*g*-PAAm were first dried under a stream of air and then inside a vacuum oven at 40 °C. Cellulose-*g*-PHEAAm was filtrated out from the polymerization mixture, washed with cold water, and lyophilized.

Typical film preparation: Both cellulose dissolution and film preparation are performed by following the procedures introduced in our previously reported work, with small changes [28]. Briefly, the ABIL was prepared by adding first TMG (14 mL, 0.11 mol) to a round bottom flask and heated at *T* = 90 °C. Then, acetic acid (HOAc) was slowly added under stirring to TMG, to achieve a 1:1 molar ratio mixture. To reduce the viscosity, 10 mL of DMSO was added to the mixture as a co-solvent. Finally, cellulose (H or M)-*g*-PNIPAAm (~1.8 g) was added to the solvent under vigorous stirring and left for 2 h at *T* = 90 °C, to obtain a 6 wt.% cellulose-*g*-PNIPAAm solution. Cellulose-*g*-PNIPAAm solutions were dropped on a glass plate and spread with a micrometric film applicator (1000 µm, Zehntner, ZUA 2000 series) and then placed into ethanol (cellulose solution: ethanol = 1:10 vol%) for regeneration, washed five times, 30 min for each washing. The use of water for regeneration led to films fragmentation. However, the use of ethanol does not allow an effective ABIL removal from the film. Therefore, to obtain films, mixture of celluloses were dissolved, using cellulose pulp as our recent work [28] and cellulose-*g*-PNIPAAm (50/50 wt.%), to obtain a 6 wt.% solution. Then, the procedure was similar, but spread solution was placed into water regeneration bath for a better ABIL removal. Finally, regenerated films materials were dried in a ventilated oven at *T* = 60 °C for 12 h.

ABIL quantification: The amount of ABIL immobilized in film was calculated by adapting the method developed in our previous work [28]. Briefly, final film was extracted into deuterium oxide (D_2_O), for 24 h, at room temperature. The resulting solution was analyzed by ^1^H-NMR, using DMF as an internal standard. The amount of ionic liquid present was calculated by relating NMR peaks area of DMF and acetic acid.

## 3. Results and Discussion

### 3.1. Cellulose-BriB Preparation and Electrochemical Characterization

**Scheme 3:**
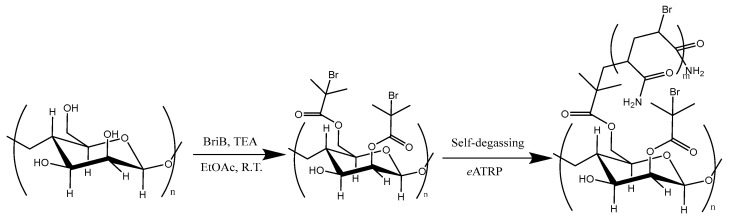
Flowchart of our approach: (1) heterogeneous modification of cellulose with 2-bromoisobutyryl bromide (BriB) dispersed in ethyl acetate in the presence of triethylamine as base. (2) *se*ATRP to grow poly(*N*-alkylacrylamides). Acrylamide (AAm) is chosen as representative monomer for the polymerization step.

We chose -BriB as a general and versatile initiation site for acrylates, styrenes, (meth)acrylamides, and sometimes methacrylates (Scheme 3) [32].

Esterification with 2-BriB occurs whether the cellulose is dissolved or dispersed. Mixtures such as DMF/LiCl, ionic imidazolium liquids [AMIm][Cl], superbases, NMP, and other hydrogen network disruptors are used to solubilize and functionalize cellulose [33,34,35] but may also be incompatible with TEA or 2-BriB during esterification, leading to undefined reactions and materials. They can also lead to lengthy and costly purifications that are not suitable for providing materials in large quantities. When cellulose is functionalized in dispersion, a dispersing “solvent” is required and typically is chloroform or dichloromethane, but chlorinated solvents should be abandoned soon [36,37]. We use ethyl acetate, a non-protic and greener alternative [38,39], but rarely used for this reaction and to our knowledge, not yet used for the functionalization of cellulose in dispersion. Since we anticipated that our scaled reactions would require non-negligible amounts of material per run, we adapted this synthesis to produce up to 15 g of cellulose-BriB per batch of esterification. Two modified cellulose-BriB starting substrates are produced, differing in the amount of BriB used (Scheme 3 and Table 1).

**Figure 1:**
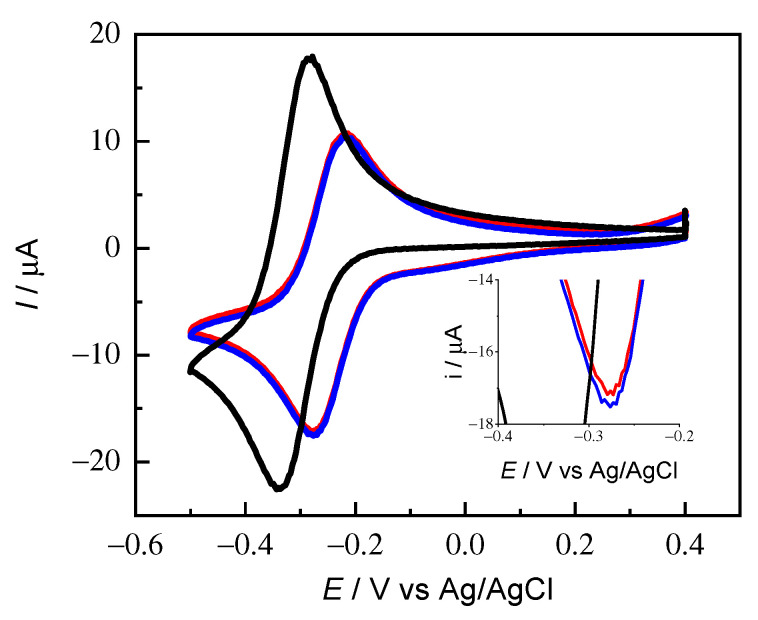
Cyclic voltammetry of [Cu^II^TPMA]^2+^ in H_2_O + 0.1 M NaBr (▬), and in the presence of 10 vol% OEOMA_500_ (▬), and in the presence of (▬) cellulose(H)-BriB recorded at *T* = 25 °C on a GC electrode at 0.2 V/s.

The presence of BriB-initiating groups on the cellulose surface was detected by cyclic voltammetry. Due to their dispersed nature and very limited diffusion, we expected a low catalytic current. We therefore decided to use [Cu^II^TPMA]^2+^ (TPMA = tris(2-pyridylmethy)lamine) and the very reactive hydrophilic methacrylate OEOMA_500_ (oligo(ethylene glycol)_500_ methyl ether methacrylate) to measure the catalytic current (Figure 1). This catalyst in H_2_O has a very high *k*_act_ for BriB (>10^6^ M^−1^s^−1^) [40] and in its presence and in the presence of OEOMA_500_, we would detect (small) catalytic currents. 

[Cu^II^TPMA]^2+^ in H_2_O + 0.1 M NaBr exhibits a reversible CV as both [Cu^II^TPMA]^2+^ and [Cu^I^TPMA]^+^ are stable in the time scale of the measurement (Figure 1). After addition of OEOMA_500_, the catalyst signal shifted to a more positive potential as the environment became more “organic”. This is a known behavior of copper catalysts once the monomer is added, and [Cu^I^L]^+^ becomes a weaker (less active) reducing agent in water/monomer mixtures [18,40,41]. Indeed, the monomer is always present in considerable amounts in the ATRP mixture. No catalytic current is observed in the presence of acrylamide (Figure S1). Finally, when the substrate (cellulose-BriB) is added, a small catalytic current is measured, since the catalyst is reduced from [Cu^II^TPMA]^2+^ to [Cu^I^TPMA]^+^, it diffuses away from the electrode and activates a BriB group, turning back to the deactivator [Cu^II^TPMABr]^+^. The deactivator diffuses back to the surface of the electrode and the process repeats [17]. However, because the cellulose-BriB macroalkyl halide is dispersed, the catalytic current captured is small. In any case, its presence indicates that: (1) esterification effectively generates a suitable cellulose-based initiator; (2) typical ATRP catalysts, once (re)generated, can activate the initiator site on cellulose; and (3) activation occurs even when the cellulose is dispersed. On the other hand, the reduction potential of alkyl bromides in water/monomer mixtures is more negative than in pure water [40]. Therefore, the presence of monomers in the reaction medium makes reduction of alkyl bromides by [Cu^I^L]^+^ less favorable than in water. The considerable effect of the monomer toward [Cu^I^L]^+^ activity can be explained by the existence of a significant interaction between the monomer and [Cu^I^L]^+^ [42]. This interaction can be observed in the voltametric response of [Cu^II^L]^2+^ in the presence of acrylamides, which is not completely reversible, supporting the presence of a rather strong interaction between the monomer and Cu^I^ (Figure S1). Unfortunately, [Cu^II^TPMA]^2+^ is not the best catalyst for the polymerization of acrylamides [10,43,44,45,46,47] and the more active [Cu^II^Me_6_TREN]^2+^ is necessary. This change is positive for the polymerization as the activation of cellulose-BriB is even faster [40]. On the other hand, (re)activation of poly(*N*-alkylacrylamide)-Br chain-end is more difficult [40]. As anticipated, the catalytic current is not visible in the presence of the substrate, despite the use of [Cu^II^Me_6_TREN]^2+^ (Figure S1).

### 3.2. Low Volume Polymerizations

To fully exploit our background knowledge of scale-up, we favored aqueous dispersion polymerizations of cellulose-BriB with acrylamide (AAm), *N*-hydroxyethylacrylamide (HEAAm), and the thermoresponsive *N*-isopropylacrylamide (NIPAAm) because of their relatively rapid and controlled polymerization in aqueous ATRP [10,14,43,44,45,47]. In addition, these polymers also find applications in industry: polyacrylamide is a water-soluble, non-toxic polymer that is widely used as a flocculant in water treatment; PNIPAAm is a water-soluble polymer that exhibits LCST and provides smart, temperature-sensitive materials; PHEAAm is an amphipathic polymer that is soluble in both aqueous and organic solvents. ATRP of acrylamides requires low temperatures (*T* = 0–10 °C) to avoid unwanted intramolecular cyclization that destroys the chain-end functionality. Small-scale polymerizations were performed at *T* = 0 °C while those at 500 mL or 1000 mL were instead performed at *T* = 10 °C [10,43,45,46,47,48]. There are no real differences between a *se*ATRP of acrylamide at 0 and 10 °C. In this work, we followed the same optimization reported in earlier scale-up reports, where we opted to run the reactions at a small scale at 0 °C while the scale-up at 10 °C, because it easier to cool and maintain the temperature at 10 °C than at 0 °C.

**Figure 2:**
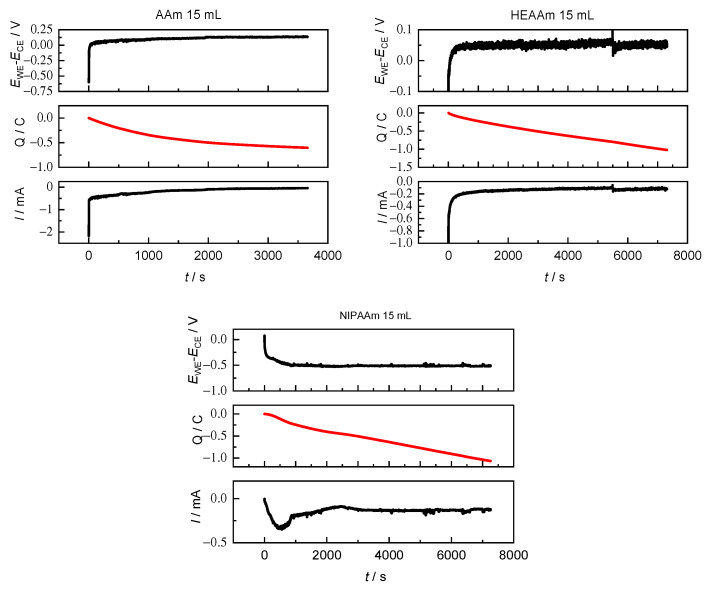
Profiles of *E*_WE_-*E*_CE_, Q, and *I* vs. time recorded during the potentiostatic *se*ATRP of 10 wt% AAm, HEAAm, and NIPAAm in H_2_O +0.1 M NaBr + 0.05 M SP at *T* = 0 °C. *E*_app_ = *E*_pc_. Working electrode = Pt mesh, counter electrode = Al wire. Both WE and CE have ~6 cm^2^ surface area. *V =* 15 mL. Substrate is cellulose(H)-BriB.

Unlike generally used molecular initiators, cellulose-BriB is completely insoluble because esterification with 2-BriB does not affect the macroscopic properties of cellulose. This may seem limiting at first glance, but this insolubility could also facilitate the separation and recovery of the material after polymerization. It also simplifies reaction preparation: cellulose-BriB is simply dispersed in H_2_O along with the other reactants (Table 2). To make the polymerization self-degassing, 0.05 M sodium pyruvate is added as a scavenger to suppress ROS formed during polymerization and keep the O_2_ content at an inert level [10,14]. Table 2 shows the typical composition of a polymerization mixture:

The scale-up protocol is practically the same of our earlier works on the scale-up of acrylamides by *se*ATRP [10]. Briefly, the polymerizations were first optimized at a small scale (15–40 mL), then scaled up to 500–1000 mL. The results of the polymerizations at 15 mL are shown in Table 3:

From the electrochemical point of view, these potentiostatic polymerizations consumed a very low amount of charge (Figure 2) and the cell tension (*E*_WE_-*E*_CE_) was low as well. This further suggested to us that the charge consumed by the scale-up reactions would be relatively low.

In a relatively short time, polymerization reached medium to high conversions. The conversions are similar for NIPAAm and AAm, while the conversion is higher for HEAAm. *Đ* shows that the cleaved polymers have *Đ* = 1.15–1.50. We also tried to obtain suitable polymerizations with cellulose (L) substrate (Table 3, entry 2) and AAm as the monomer, but the conversion reached only 40% and *Đ* was 1.50. As the small-scale results were promising, we sought the possibility to proceed immediately to higher scales.

### 3.3. Large Volume Polymerizations

**Figure 3:**
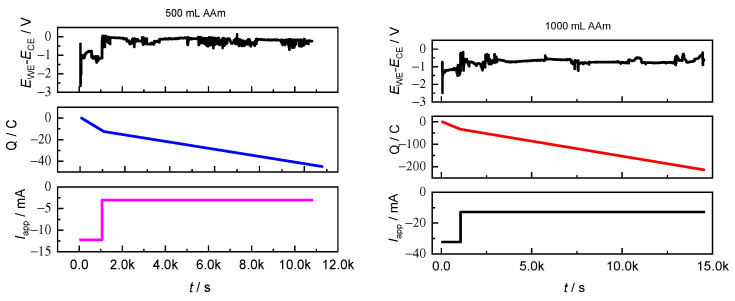
Profiles of *E*_WE_-*E*_CE_, Q, and *I*_app_ vs. time recorded during the galvanostatic *se*ATRP of 10 wt% AAm in H_2_O + 0.05 M NaBr + 0.05 M SP at *T* = 10 °C. *V* = 500 and 1000 mL, respectively.

**Figure 4:**
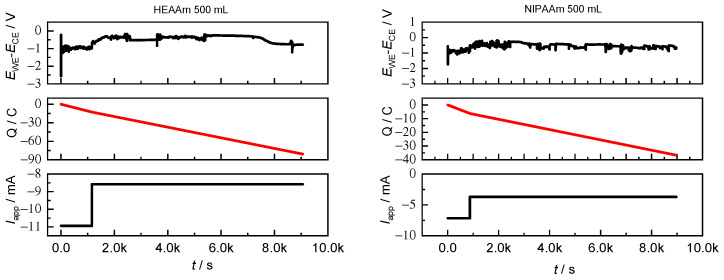
Profiles of *E*_WE_-*E*_CE_, Q, and *I*_app_ vs. time recorded during the galvanostatic *se*ATRP of 10 wt% HEAAm and NIPAAm in H_2_O + 0.05 M NaBr + 0.05 M SP at *T* = 10 °C. *V* = 500 mL.

We decided to introduce galvanostatic *se*ATRP for all reactions above 15 mL [45,47]. Exceeding this volume, all reactions were driven by two-step galvanostatic electrolysis, deriving *I*_app_ values knowing: (1) the charge passed (Q); (2) the chronoamperometry profiles recorded at 15 mL scale (potentiostatic *se*ATRP); (3) the targeted polymerization volume; and (4) the geometric properties of the reactors, similar to previous works [10]. Details of the electrolysis programs are shown in Table 4:

From the electrochemical point of view, the galvanostatic polymerizations were constrained by the scaled *I*_app_ values, allowing the regeneration of [Cu^I^Me_6_TREN]^+^ in a simpler electrochemical setup. Profiles of *E*_WE_-*E*_CE_, Q, and *I*_app_ recorded during large-scale polymerizations are shown in Figure 3 (for Aam at 500 and 1000 mL) and Figure 4 (for HEAAm and NIPAAm at 500 mL):

Additional electrochemical profiles at 40 mL volume are shown in Figures S2–S4. At 40 mL, the polymerization reached moderate to high conversions in a relatively short time, producing cellulose-poly(*N*-alkyl)acrylamide-Br materials in a similar manner as at 15 mL. Conversions were higher for polymerizations initiated with the cellulose(H)-BriB substrate. The most controlled polymer for both substrates is PNIPAAm. Encouraged by these results, the polymerization volume was further increased. At 500 mL, the conversions of NIPAAm and HEAAm are highest, and a near quantitative conversion is observed for HEAAm (Table 5, entry 8). Representative ^1^H-NMR of samples taken during the polymerizations at this volume are shown in Figures S5–S7. The most controlled polymer at 500 mL is again PNIPAAm. A *se*ATRP was performed on a 1000 mL scale using Aam. Unfortunately, PAAm-Br is poorly controlled in this case. It is likely that the uncontrolled growth is due to side reactions that occur in the later stages of polymerization when the monomer concentration decreases and the potential drifts towards more negative values to match *I*_app_. This can lead to excessive regeneration of the catalyst or even to its over-reduction to Cu^0^. We have indeed observed some Cu^0^ deposits on the walls of the reactor due to overreduction. This shows that more current/time steps are needed for volumes above 500 mL (at least for AAm). Likely, the scale of the reaction can be increased beyond 1 L, with additional current steps. 

### 3.4. Materials Characterization

IR spectra, GPC, SEM, TGA, and DSC of the synthetized materials confirmed the presence of polymers onto cellulose.

#### 3.4.1. FTIR Spectra of Cellulose and Its Synthesized Cellulose-Based Materials

**Figure 5:**
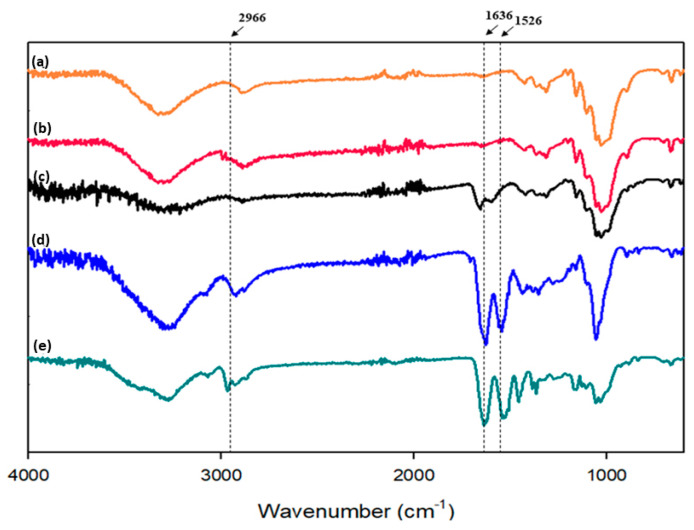
IR spectra of different substrates. (a) MCC; (b) cellulose(H)-BriB; (c) cellulose(H)-*g*-PAAm-Br; (d) cellulose(H)-*g*-PHEAAm-Br; and (e) cellulose(H)-*g*-PNIPAAm-Br.

In Figure 5, the hydroxyl group (-OH) stretching vibrations near 3300 cm^−1^ correspond to both water absorption and cellulose hydroxyl groups [25,28]. After *se*ATRP, the grafted poly(*N*-alkyl)acrylamides (Figure 5c–e), showed new peaks at 1636 cm^−1^ and 1526 cm^−1^ corresponding to the amide I and amide II stretching vibrations. In the case of PNIPAAm, the peak at 2966 cm^−1^ corresponds to the C–H stretch of the isopropyl group appear [49], thus confirm the successful growth of PNIPAAm on the substrate.

#### 3.4.2. GPC Chromatograms of Cleaved poly(*N*-alkyl)acrylamide-Br Chains from Grafted Cellulose

**Figure 6:**
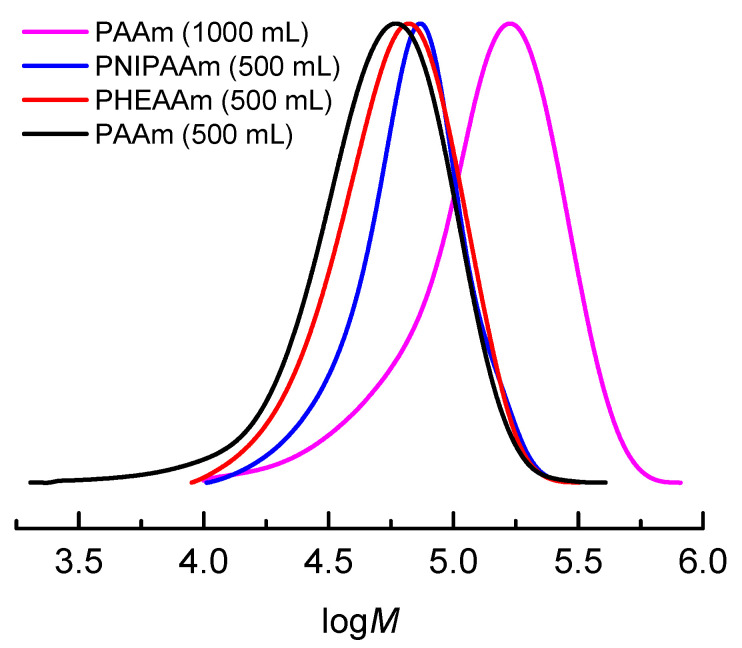
GPC chromatograms of poly(*N*-alkyl)acrylamide-Br cleaved from cellulose surface after the galvanostatic *se*ATRP at 500 mL: PAAm 500 mL (▬), PHEAAm 500 mL (▬), PNIPAAm 500 mL (▬), and PAAm 1000 mL (▬).

GPC chromatograms (Figure 6, additional chromatograms showing the cumulative weight fraction, WF/*d*LogM, and the normalized weight fraction are shown in Figures S15–S18 of cleaved poly(*N*-alkyl)acrylamide-Br prepared at the 500 mL scale show monomodal molecular weight distributions. All poly(*N*-alkyl)acrylamides prepared at the 500 mL scale have *Đ*~1.16–1.60, with PAAm-Br having the highest *Đ*. PAAm-Br prepared at the 1000 mL scale has even worse *Đ* and *M*_n_, which is significantly higher than the *Đ* of all other poly(*N*-alkyl)acrylamide Br prepared at the 500 mL scale.

#### 3.4.3. Thermal Analysis of Cellulose-poly(*N*-alkyl)acrylamide-Br Materials

**Figure 7:**
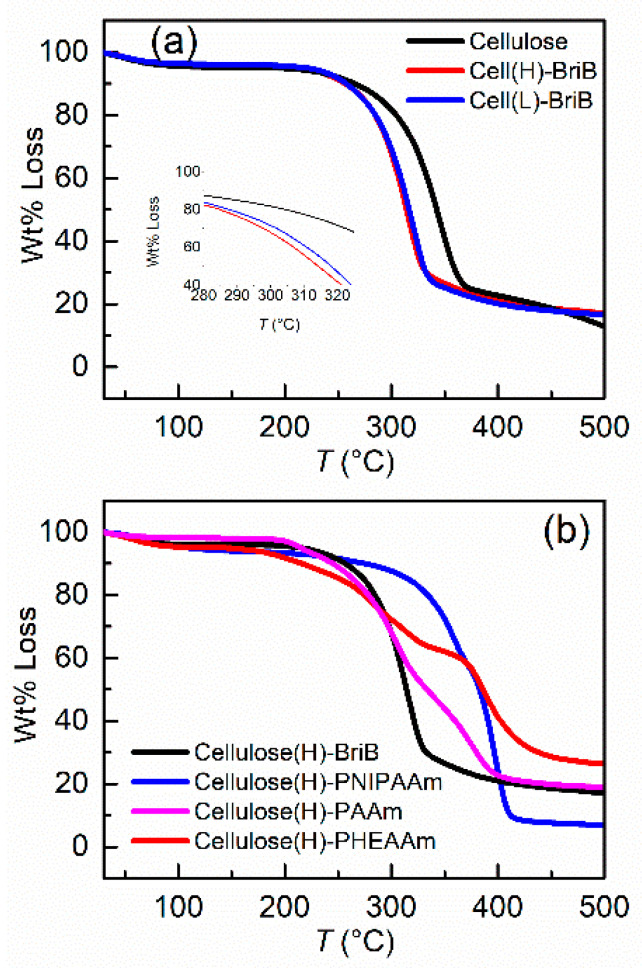
TGA analysis of (**a**) pristine MCC (▬), cellulose(H)-BriB (▬), and cellulose(L)-BriB (▬); (**b**) cellulose(H)-BriB before (▬) and after the polymerization of NIPAAm (▬), HEAAm (▬), and AAm (▬).

**Figure 8:**
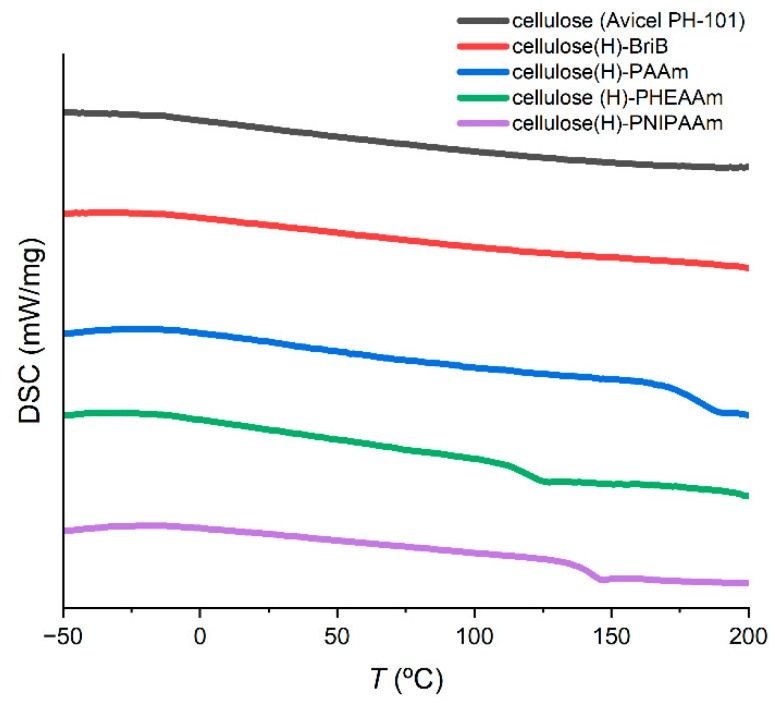
DSC thermograms of MCC (▬), cellulose(H)-BriB (▬), cellulose(H)-PAAm-Br (▬), cellulose(H)-PHEAAm-Br (▬), and cellulose(H)-PNIPAAm-Br (▬) recorded from *T* = −50 °C to *T* = 200 °C.

Thermal stability of MCC, cellulose-BriB, and cellulose-*g*-poly(*N*-alkylacrylamide)-Br are evaluated by TGA (Figure 7):

Thermal stability is affected by both esterification with 2-bromoisobutyryl bromide and by the growth of polymers on the substrate (Figure 7). The slight decrease in thermal stability caused by esterification is observed above 250 °C but despite the different amounts of 2-BriB used, the substrates have comparable thermal stability. After *se*ATRP, the thermal stability of the materials increased and the onset of degradation is observed above 316 °C. This increase is due to the presence of poly(*N*-alkyl)acrylamides, which have higher thermal stability than cellulose-BriB [50,51]. The typical two-step degradation profile of acrylamides can also be seen, especially in the case of cellulose-*g*-PHEAAm-Br [52]. The thermal transitions of these materials were also investigated using differential scanning calorimetry (DSC). Figure 8 shows the thermograms recorded:

However, after the polymerizations, the observed glass transition temperatures (*T*_g_) agree with literature values. The *T*_g_ values seen in the thermograms are characteristic of the corresponding polymers: *T*_g_ = 185 °C for PAAm [53], *T*_g_ = 101 °C for PHEAAm [54], and *T*_g_ = 141 °C for PNIPAAm [55]. This also confirms the growth of poly(*N*-alkyl)acrylamides-Br at initiation sites located at cellulose chains

#### 3.4.4. Film Preparation and Characterization by Contact Angle Measurements

**Figure 9:**
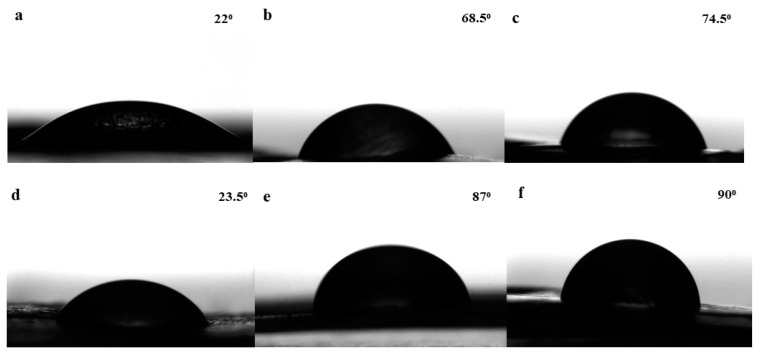
Contact angle measurement at *T* = 25 °C (**a**–**c**) and at *T* = 50 °C (**d**–**f**) of (**a**,**d**) MCC control, (**b**,**e**) cellulose(L)-PNIPAAm, and (**c**,**f**) cellulose(H)-PNIPAAm.

Among the prepared materials, we chose cellulose-PNIPAAm-Br prepared with cellulose-BriB (H = high and L = low) to make thermoresponsive films. PNIPAAm is a temperature sensitive and biocompatible polymer with a lower critical solution temperature (LCST) in H_2_O of ∼32 °C [56]. This distinguishing thermoresponsiveness is widely exploited in the design of controlled drug delivery systems [57], tissue engineering [58], and biosensing [59]. The process of film formation is the same as that described in a previous work with [TMG][OAc] ABIL [28]. However, when the cellulose-*g*-PNIPAAm-Br solution entered the regeneration bath with water (at *T* < LCST), the regenerated material could not maintain its shape and consistency and broke apart. Changing the regeneration bath from water to ethanol resulted in better regeneration of the material, maintaining both shape and consistency. However, this also resulted in less leachability of [TMG][OAc], as ethanol is less prone to remove this ABIL. This resulted in sticky and yellowish films due to the high content of [TMG][OAc]. In each case, the thermoresponsiveness of the films was evaluated by contact angle measurements at *T* = 25 °C (below LCST) and at *T* = 50 °C (above LCST). Figure 9 shows the effect of the presence of PNIPAAm into the cellulose surface, compared also to MCC.

**Figure 10:**
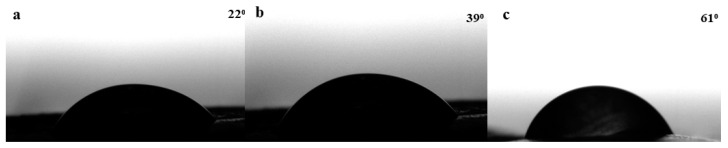
Contact angle measurement of (**a**) cellulose (MCC) control film, (**b**) Cellulose pulp + Cellulose(H)-*g*-PNIPAAm-Br film at *T* = 25 °C, (**c**) Cellulose pulp + Cellulose(H)-*g*-PNIPAAm-Br film at *T* = 50 °C.

At *T* < LCST, cellulose(H)-*g*-PNIPAAm is more hydrophobic than MCC as the contact angle increases from 24° (control sample) to ~70°. When the measurement is performed at T = 50 °C (*T* > LCST), the contact angle of the cellulose(H)-PNIPAAm-Br samples additionally increases to 90°, while the contact angle of the control sample is maintained. This confirms the thermoresponsiveness of the cellulose(H)-PNIPAAm-Br material. The thermoresponsiveness is confirmed, but due to the presence of ABIL, the film formation process needs further improvement. To obtain a consistent material that can be regenerated in a water bath, a different approach was used and cellulose(H)-PNIPAAm-Br was dissolved with cellulose pulp as in our previous work [28]. Fortunately, an equal mass ratio of cellulose(H)-PNIPAAm-Br and cellulose pulp allowed regeneration in water, maintaining both shape and stability as well as a solvent-free film. Here, cellulose pulp served as the matrix for the composite of the cellulose-PNIPAAm-Br material. The resulting composite was further characterized by IR, SEM, DSC, TGA, and analyzed again by contact angle measurement (Figure 10).

When cellulose(H)-*g*-PNIPAAm-Br was mixed with 50 wt% cellulose pulp (Figure 10b), the contact angle increased from 22° to 39° compared to the control sample (Figure 10a). When the film was heated to T = 50 °C, the contact angle also increased to 61°, proving the thermoresponsiveness of the resulting film. The value obtained is lower than for the pure cellulose PNIPAAm samples (Figure 9), which was expected due to the higher cellulose content in the final films. This confirms the thermoresponsive behavior of PNIPAAm: at T > LCST, the material becomes more hydrophobic. The behavior of the film can be switched from hydrophilic to hydrophobic by increasing T above LCST [60], and can be manipulated by changing the mass content of the individual components in the film. 

#### 3.4.5. Infrared Spectroscopy of Composite Films (FTIR)

**Figure 11:**
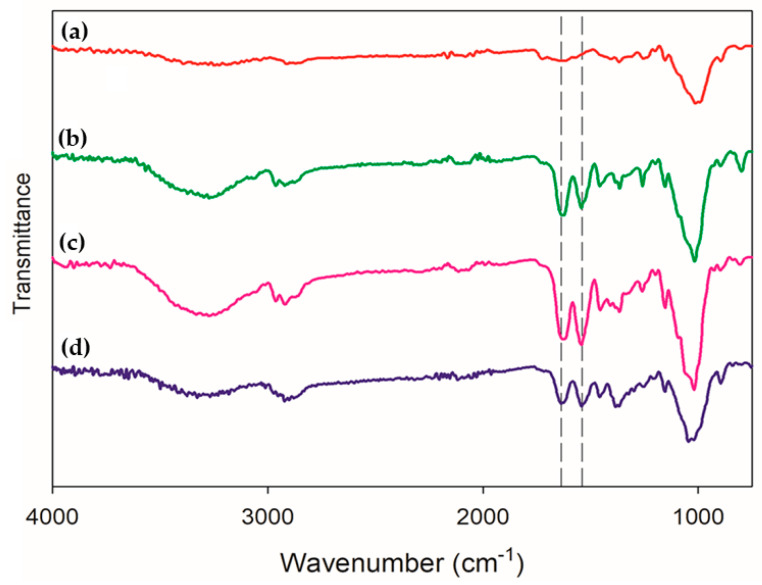
FTIR spectra of different cellulose films (a) MCC film, (b) cellulose(L)-*g*-PNIPAAm-Br, (c) cellulose(H)-*g*-PNIPAAm-Br, and (d) 50 wt% cellulose(H)-*g*-PNIPAAm-Br + 50 wt% cellulose pulp.

FTIR spectra of cellulose-*g*-PNIPAAm-Br films (Figure 11) show the characteristic peaks of cellulose and PNIPAAm; no new absorption peaks appeared, suggesting that there is no chemical reaction between cellulose(L or H)-*g*-PNIPAAm-Br and [TMG][OAc] [28]. The presence of PNIPAAm is confirmed by peaks at 1636 cm^−1^ and 1526 cm^−1^ corresponding to the stretching vibrations of amide I and amide II of PNIPAAm. When the process of film formation with cellulose pulp was adjusted, the washability of the films with water was also enabled.

#### 3.4.6. Extraction Test

However, FTIR does not readily confirm this fact, as the typical peaks of the ABIL solvent are absent once its typical peaks at 1543 and 1380 cm^−1^ are in the same range as the characteristic peaks of PNIPAAm. An extraction test developed in our previous work was performed to confirm the absence of ABIL in the resulting film [28].

The water used in the washing step promotes cellulose regeneration by displacing [TMG][OAc]. Quantification of ABIL in the final film was carried out by using our previous method [28], as the residual peak of TMG is not visible at this time, the integrals of the two methyl groups of the acetate anion (~2.22 ppm) are used to calculate the residual [TMG][OAc] (Figure S8). If the molar ratio remains unchanged [25], the mass of each component is then calculated. Table 6 shows the quantification of [TMG][OAc]:

Note that the mass of [TMG][OAc] extracted in the test was not the total mass weighted in the NMR tube once the test is performed in 2 mL of D_2_O and only 700 µL were used in this test. The mass extracted for the 2 mL solution is therefore extrapolated (Table 7).

As can be seen, the resultant composite film has only a residual amount of the solvent [TMG][OAc].

#### 3.4.7. Scanning Electronic Microscopy (SEM)

**Figure 12:**
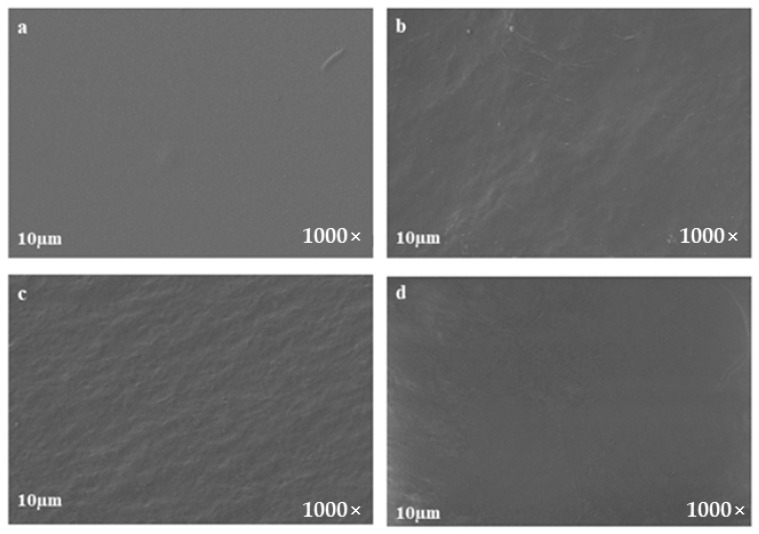
SEM images of cellulose(H)-*g*-PNIPAAm-Br films: (**a**) cellulose pulp film; (**b**) cellulose(M)-*g*-PNIPAAm-Br film; (**c**) cellulose(H)-*g*-PNIPAAm-Br film; (**d**) 50 wt% cellulose pulp + 50 wt% cellulose(H)-*g*-PNIPAAm-Br film. All images were taken at a ×1000 magnification.

SEM analysis showing morphology of the materials recovered after the polymerization is shown in Figures S8–S10. The morphology of the resulting films was analyzed using SEM (Figure 12). As expected, the cellulose control film (Figure 12a) exhibits a homogeneous and smooth surface [25]. As the amount of PNIPAAm increases (Figure 12b,c), the surface roughness increases, which is probably due to the temperature used in drying the films when the films are heated to *T* = 60 °C, since PNIPAAm is thermosensitive. For films with unmodified pulp, the formed film showed a homogeneous and smooth surface (Figure 12d) as a control sample. Possibly, the lower amount of PNIPAAm in these samples as well as the presence of pulp led to a smooth surface resulting from a uniform distribution of the individual cellulose components.

## 4. Conclusions

Suitable cellulose-grafted poly(*N*-alkyl)acrylamide materials are obtained by an enlarged version of self-degassing *se*ATRP in dispersion with a reaction volume of up to 1000 mL. Using a commercial MCC substrate esterified with 2-bromoisobutyryl bromide (2-BriB), various poly(*N*-alkyl)acrylamides were grown on cellulose by self-degassing dispersion *se*ATRP in water. Cellulose-grafted poly(*N*-alkyl)acrylamide-Br materials were characterized by FTIR, SEM, thermally by TGA/DSC, and contact angle. Cellulose-*g*-PNIPAAm-Br is the simplest material that can be obtained from the exhaust gas working solution by simple heating, thanks to its thermosensitivity. This material was chosen for the preparation of cellulose-based films by dissolving it in [TMG][OAc] ABIL. Regeneration in a water bath was not possible, resulting in ABIL contamination in the films. A new formulation in which the grafted polymer was mixed with native cellulose pulp gave the new film more consistency and stability and allowed regeneration in a water bath and ABIL washability. The resulting films were characterized by FTIR, SEM, and TGA/DSC analysis. Contact angle measurements showed that the films were thermoresponsive even when the PNIPAAm substrate was mixed with raw pulp. These results demonstrate just one aspect of the multi-faceted versatility of cellulose as a building block, and that spiked versions of self-degassing *se*ATRP can contribute significantly to the broader use of virgin or recycled cellulose as a feedstock to produce cellulose polymer materials. This self-degassing *se*ATRP in dispersion conditions could be used in the future not only for acrylamides but also for hydrophobic monomers.

## Figures and Tables

**Table 1:** Cellulose-BriB macro(alkyl halides) prepared by esterification of MCC with 2-BriB in EA/TEA at room temperature.

Macroinitiators	Cellulose Starting Amount (g)	BriB/TEA/EA (mL)	Material Recovery (%)
**Cellulose (H)-BriB**	15	16.1/18.2/400	99
**Cellulose (L)-BriB**	15	8.0/9.1/400	98

**Table 2:** Chemical composition mixture of the candidate polymerization up to scale-up factor ~67.

Name	Empirical Formula	Role	Concentration	Quantity (at 500 mL)
Acrylamide (AAm), 2-hydroxyethyl acrylamide (HEAAm), N-isopropylacrylamide (NIPAAm)	C_3_H_5_NO (AAm) C_5_H_9_NO_2_ (HEAAm) C_6_H_11_NO (NIPAAm)	Monomers	1.41 M (AAm) 0.96 M (HEAAm) 0.88 (NIPAAm)	50 g (AAm) 25 g (HEAAm) 25 g (NIPAAm)
Water	H_2_O	Solvent	~50 M	450 mL
Copper (II) Bromide	CuBr_2_	Precatalyst	5 × 10^−4^ M	111.65 mg
Tris(2-dimethylaminoethyl) amine (Me_6_TREN)	C_12_H_30_N_4_	Ligand	2 × 10^−3^ M	460.8 mg
Sodium Pyruvate (SP)	C_3_H_3_O_3_Na	ROS scrubber-excitant	5 × 10^−2^ M	2.75 g
Buffer	Na and K Phosphates	Buffer components	10mM	709.6 mg (Na) 122.5 mg (K)
Sodium Bromide	NaBr	Halide salt	5 × 10^−2^–10^−1^ M	5.124 g
Dimethylformamide (DMF)	C_3_H_7_NO	NMR internal standard	2 vol%	10 mL
Cellulose-BriB	(C_6_H_1_BrO_3_)_n_-(C_4_H_7_BrO2)_m_	Macroalkyl halide initiator	1.5–3 wt%	7.5 g

**Table 3:** Self-degassing potentiostatic *se*ATRP of Aam, HEAAm, and NIPAAm (all 10 wt%) starting from cellulose-BriB (H = high, L = low) in the presence of 0.05 M SP as ROS scavenger and 0.1 M NaBr as supporting electrolyte. Reaction volume is 15 mL. *^a^*.

Entry	Monomer	Cellulose Type	*E*_app_	*t* (h)	Conv. (%) *^b^*	*M*_n_^app *c*^ (kg/mol)	*Đ ^d^*	|*Q*| (C)
**1**	AAm	H	*E*_pc_	1	67	38.1	1.45	0.60
**2**	AAm	L	*E*_pc_	1	40	30.9	1.50	0.69
**3**	NIPAAm	H	*E*_pc_	2	69	33.3	1.15	2.42
**4**	HEAAm	H	*E*_pc_	2	80	35.6	1.44	1.07

*^a^*^.^ Conditions: [AAm]/[CuBr_2_]/[Me_6_TREN]/[NaBr] = 704/1/4/100, [NIPAAm]/[CuBr_2_]/[Me_6_TREN]/[NaBr] = 884/0.1/0.4/10, [HEAAm]/[CuBr_2_]/[Me_6_TREN]/[NaBr] = 869/0.1/0.4/10; WE = Pt mesh approx. 6 cm^2^, CE = aluminum wire directly immersed into the working solution. Stirring = 700 rpm. *E*_pc_ = cathodic peak potential. *^b.^* Calculated from ^1^H-NMR in D_2_O + 2 vol% DMF as internal standard. *^c.^* Calculated from aqueous GPC with six narrow poly(sodium methacrylate) standards at *T* = 35 °C except for entry 3 from GPC in DMF +0.03 wt% LiBr with six narrow poly(methyl methacrylate) standards at *T* = 60 °C. *^d.^* *Đ* = *M*_w_/*M*_n_.

**Table 4:** Electrolysis programs, geometrical reactors details for the candidate polymerization up to 1000 mL (scale-up factor ~67). *^a^*.

Volume (V_f_)	Monomer	Cellulose Type	Step	Current Intensity (mA)	Time (s)	|*Q*_erogated_| (C)	Liquid Height (cm)	Surface (cm^2^)	*S*/*V* (cm^−1^)
40	AAm	H and L	1	−0.790	870	0.687	3.3	53.09	0.753
40	AAm	H and L	2	−0.386	6330	2.445			
**Total**					7200	3.312			
40	NIPAAm	H and L	1	−0.790	870	0.687	3.3	53.09	0.753
40	NIPAAm	H and L	2	−0.386	4500	1.737			
**Total**					5300	2.424			
40	HEAAm	H and L	1	−1.135	1160	1.316	3.3	53.09	0.753
40	HEAAm	H and L	2	−0.891	6060	5.399			
**Total**					7220	6.716			
500	AAm	H	1	−12.227	1060	12.54	3.6	302.35	0.605
500	AAm	H	2	−3.043	9740	32.39			
**Total**					10,800	44.93			
500	HEAAm	H	1	−10.94	1160	12.69	3.6	302.35	0.605
500	HEAAm	H	2	−8.576	7840	67.24			
**Total**					9000	79.93			
500	NIPAAm	H	1	−7.162	870	6.23	3.6	302.35	0.605
500	NIPAAm	H	2	−3.710	7840	29.09			
**Total**					8710	35.32			
1000	AAm	H	1	−32.339	1060	34.31	6.7	444.39	0.444
1000	AAm	H	2	−12.887	13,461	173.46			
**Total**					14,521	213.77			

*^a^* Scale-up factor = *V*_f_/*V*_i_, where *V*_i_ is 15 mL.

**Table 5:** Self-degassing galvanostatic *se*ATRP of AAm, HEAAm, and NIPAAm (all 10 wt%) starting from cellulose-BriB (H = high, M = medium) in the presence of 0.05 M SP as ROS scavenger and 0.05 M NaBr as supporting electrolyte inside SS304 reactors.

Entry	Monomer	Cellulose Type	Volume (mL)	*t* (h)	Conv. (%) *^b^*	*M*_n_^app *c*^ (kg/mol)	*Đ ^d^*	|Q_erogated_| (C)
**1**	NIPAAm	L	40	2	75	64.5	1.13	3.32
**2**	HEAAm	L	40	2	58	61.3	1.36	3.32
**3**	AAm	L	40	2	55	50.3	1.44	3.32
**4**	NIPAAm	H	40	1.5	81	42.1	1.16	3.32
**5**	HEAAm	H	40	2	84	43.4	1.58	3.32
**6**	AAm	H	40	2	68	39.6	1.48	3.32
**7**	NIPAAm	H	500	2.4	90	51.3	1.16	35.32
**8**	HEAAm	H	500	2.5	98	56.5	1.29	79.93
**9**	AAm	H	500	3	86	46.2	1.57	44.93
**10**	AAm	H	1000	4	70	112.6	2.06	262.24

*^a^*^.^ Conditions entry 1, 4, and 7: [NIPAAm]/[CuBr_2_]/[Me_6_TREN]/[NaBr] = 884/0.1/0.4/5, conditions entries 2, 5, and 8: [HEAAm]/[CuBr_2_]/[Me_6_TREN]/[NaBr] = 869/0.1/0.4/5 Conditions entries 3, 6, 9–10: [AAm]/[CuBr_2_]/[Me_6_TREN]/[NaBr] = 704/1/4/50. Stirring = 300 rpm. *^b.^* Calculated from ^1^H-NMR in D_2_O + 2 vol% DMF as the internal standard. *^c.^* Calculated from aqueous GPC with six narrow poly(sodium methacrylate) standards at *T* = 35 °C, except for entries 1, 4, and 7 from GPC in DMF +0.03 wt% LiBr with six narrow poly(methyl methacrylate) standards at *T* = 60 °C. *^d.^ Đ* = *M*_w_/*M*_n_.

**Table 6:** Quantification of [TMG][OAc] extraction by ^1^H-NMR spectrum analysis.

	**Initial Volume (mL)**	**Initial Ratio (mol)**	**After Extraction**	**Relative Peak Area (NMR) *^b^***	**Proton Number *^c^***	**n × 10^−2^ (mol)**	**Mass Extraction (mg) ^*e*^**
DMF *^a^*	-	-		1.00	1	7.25	5.3
OAc	6.53	1.00		0.06	3	0.145	0.087
TMG	14	1.00		-	-	0.145 *^d^*	0.167
DMSO	10	1.26		-	-	0.183 *^d^*	0.143

*^a^* mass weighted into NMR tube: 5.3 mg; *^b^* integration value of peak in NMR spectrum; *^c^* number of protons; *^d^* assuming the same initial molar ratio; *^e^* mass of each ABIL ion calculated by molar ratio.

**Table 7:** [TMG][OAc] quantification in the cellulose pulp cellulose(H)-PNIPAAm film.

Mass Extracted (in NMR, mg)	Extrapolated Mass Extracted in 2 mL (mg)	Initial Mass of Extracted Film (mg)	ABIL Retained (wt.%)
0.397	1.134	107.1	1.1

## Data Availability

The authors confirm that the data supporting the findings of this study are available within the article and its Supplementary Materials.

## Supplementary Materials

The following supporting information can be downloaded at: https://www.mdpi.com/article/10.3390/polym14224981/s1, Sections: S1. Additional electrochemical characterizations, S2. Additional electrochemical current profiles recorded during the *se*ATRPs, S3. Additional NMR spectra, S4. Additional details of ABIL quantification, S5. SEM images of cellulose−poly(*N*−alkyl)acrylamide−Br materials. References [61,62] are cited in the supplementary materials.

## Abbreviations

AAm	Acrylamide
ABIL	Acid-base distillable ionic liquid
ATR-FTIR	Attenuated total reflectance Fourier-transform infrared
ATRP	Atom transfer radical polymerization
-BriB	2-bromoisobutyrate initiating group
CE	Counter electrode
CV	Cyclic voltammetry
*Ð*	Polydispersity (*Đ* = *M*_w_/*M*_n_)
D_2_O	Deuterium oxide (deuterated water)
DES	Deep eutectic solvents
DMF	Dimethylformamide
DMAc/LiCl	*N,N*-dimethylacetamide/lithium chloride mixtures
DMSO	Dimethyl sulfoxide
DP	Degree of polymerization (targeted)
DSC	Differential scanning calorimetry
*E*_1/2_	Half-wave potential
*E*_app_	Applied potential
*E*_WE_	Potential of the working electrode (vs. reference electrode)
*E*_CE_	Potential of the counter electrode (vs. reference electrode)
*E*_WE_-*E*_CE_	Difference of potential between working and counter electrode
*e*ATRP	Electrochemically mediated atom transfer radical polymerization
*E*_pa_	Anodic peak potential
*E*_pc_	Cathodic peak potential
*E* ^θ^	Standard redox potential
*F*	Faraday constant
GC	Glassy carbon electrode
GPC	Gel permeation chromatography
^1^H-NMR	Proton nuclear magnetic resonance
HEAAm	2-hydroxyethylacrylamide
HOAc	Acetic acid
*I*_app_	Applied current
ICAR	Initiators for continuous activator regeneration
*k*_act_	Activation rate constant
*K*_ATRP_	Equilibrium constant of ATRP
*k*_deact_	Deactivation rate constant
*k*_p_	Propagation rate constant
*k*_t_	Termination rate constant
LCST	Lower critical solubility temperature
M	(A given) monomer
[M]_0_	Initial concentration of monomer
[M]	Instantaneous concentration of monomer
MCC	Microcrystalline cellulose
Me_6_TREN	Tris [2-(dimethylamino)ethyl]amine
*M*_n_^GPC^	Number average molecular weight calculated by GPC
MW	Molecular weight
NIPAAm	*N*-Isopropylacrylamide
NMMO	*N*-methylmorpholine-*N*-oxide
-OH	Hydroxyl group
OAc	Acetate anion
PAAm	Poly(acrylamide)
(P)ICAR ATRP	(Photoinduced) ICAR ATRP
PHEAAm	Poly(*N*-hydroxyethylacrylamide)
PNaMA	Poly(sodium methacrylate)
PNIPAAm	Poly(*N*-Isopropylacrylamide)
Q	Charge passed (in Coulomb)
*R*	Universal constant of perfect gases
RDRP	Reversible deactivation radical polymerization
ROS	Reactive oxygen species
RX	Organic alkyl halide (ATRP initiators)
SCE	Saturated calomel electrode
*se*ATRP	Simplified *e*ATRP
SEM	Scanning electron microscopy
SP	Sodium pyruvate
SS304	Stainless steel 304
*T*_g_	Glass transition temperature
TGA	Thermogravimetric analysis
THF	Tetrahydrofuran
TMG	Tetramethylguanidine (and tetramethylguanidinium cation)
TPMA	Tris-[(2-pyridyl)methyl]amine
WE	Working electrode
