# Supplementary material for: Scaling-Up an Aqueous Self-Degassing Electrochemically Mediated ATRP in Dispersion for the Preparation of Cellulose–Polymer Composites and Films
Source: Polymers (Basel). 2022 Nov 17;14(22):4981. doi: 10.3390/polym14224981 (PMC9692721; doi:10.3390/polym14224981)
Supplement: Supplementary file 1 [file polymers-14-04981-s001.zip › polymers-1991114-supplementary.pdf]

Supporting Information for

Scaling-up an aqueous self-degassing electrochemically mediated ATRP in dispersion for the preparation of cellulose-polymer composites and films

Francesco De Bon,[§] Inês M. Azevedo[§], Diana C. M. Ribeiro, Rafael C. Rebelo, Jorge F. J. Coelho and Arménio C. Serra*

University of Coimbra, Centre for Mechanical Engineering, Materials and Processes, Department of Chemical Engineering, Rua Sílvio Lima, Pólo II, 3030-790 Coimbra, Portugal

[§]These authors contributed equally

**Corresponding author: aserra@eq.uc.pt*

S1. Additional electrochemical characterizations.

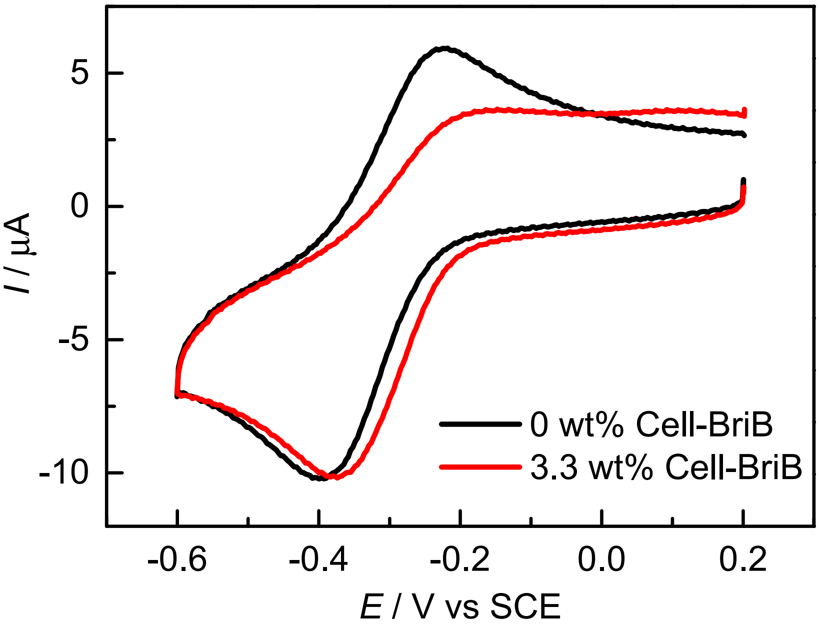

Figure S1. Cyclic voltammetry of [$Cu^{II}Me_6TREN]^{2+}$] in H_2O + 0.1 M NaBr + 0.05 M SP + 10 wt% AAm, at *T* = 0 °C. The voltammetry is recorded in the absence (▬) or in the presence (▬) of 0.5 g of cellulose(H)-BriB.

S2. Additional electrochemical current profiles recorded during the *se*ATRPs.

Additional electrochemical current, charge and E_{WE}-E_{CE} profiles are shown in **Figures S2-4**:

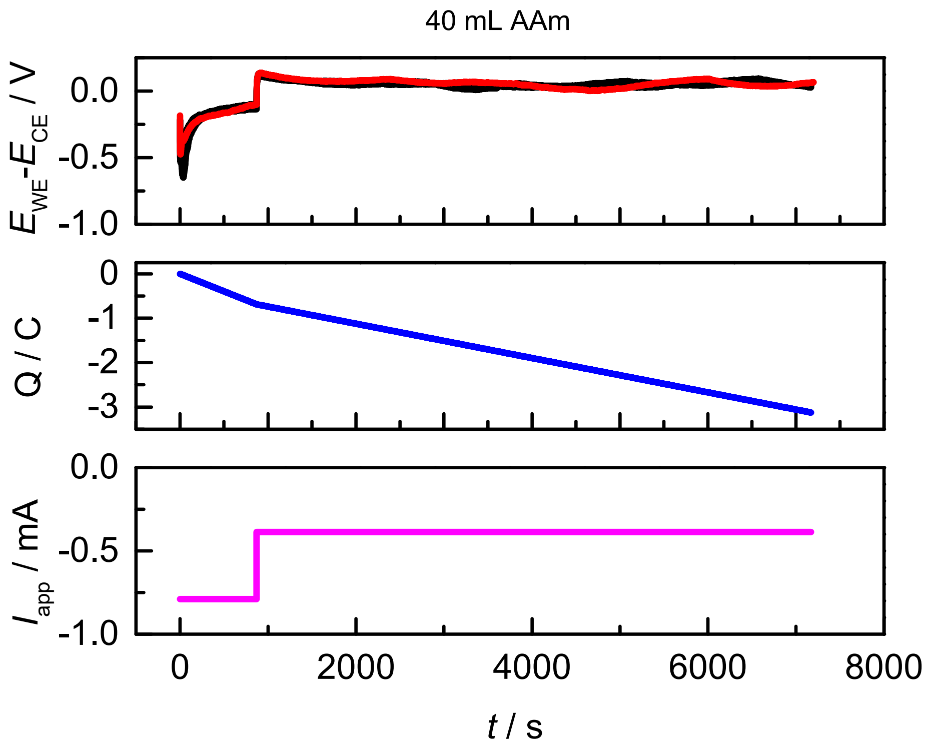

Figure S2. Profiles of E_{WE}-E_{CE}, Q and I_{app} recorded during the galvanostatic *se*ATRP of AAm 10 wt% in H_2O + 0.1 M NaBr + 0.05 M SP, in the presence of cellulose (H)-BriB (▬) or cellulose (M)-BriB (▬) at T = 0 °C. Reaction volume is 40 mL.

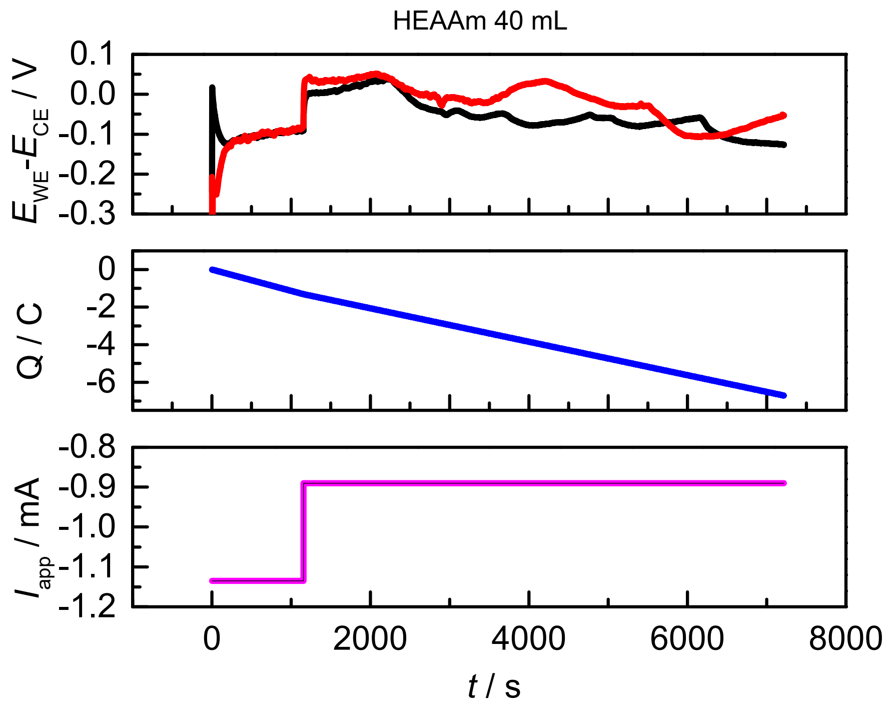

Figure S3. Profiles of E_{WE}-E_{CE}, Q and I_{app} recorded during the galvanostatic *se*ATRP of HEAAm 10 wt% in H_2O + 0.1 M NaBr + 0.05 M SP, in the presence of cellulose (H)-BriB (▬) or cellulose (M)-BriB (▬) at T = 0 °C. Reaction volume is 40 mL.

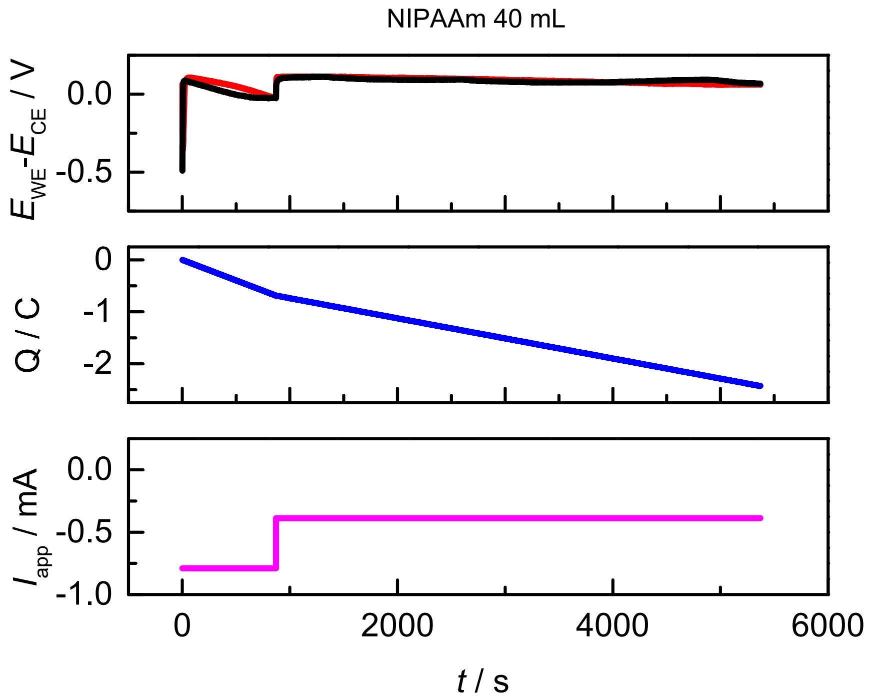

Figure S4. Profiles of E_{WE}-E_{CE}, Q and I_{app} recorded during the galvanostatic *se*ATRP of NIPAAm 10 wt% in H_2O + 0.1 M NaBr + 0.05 M SP, in the presence of cellulose (H)-BriB (▬) or cellulose (M)-BriB (▬) at T = 0 °C.

S3. Additional NMR spectra

NMR spectra recorded during the polymerizations at 500 mL scale of AAm, NIPAAm and HEAAm are shown in **Figures S5-S7**:

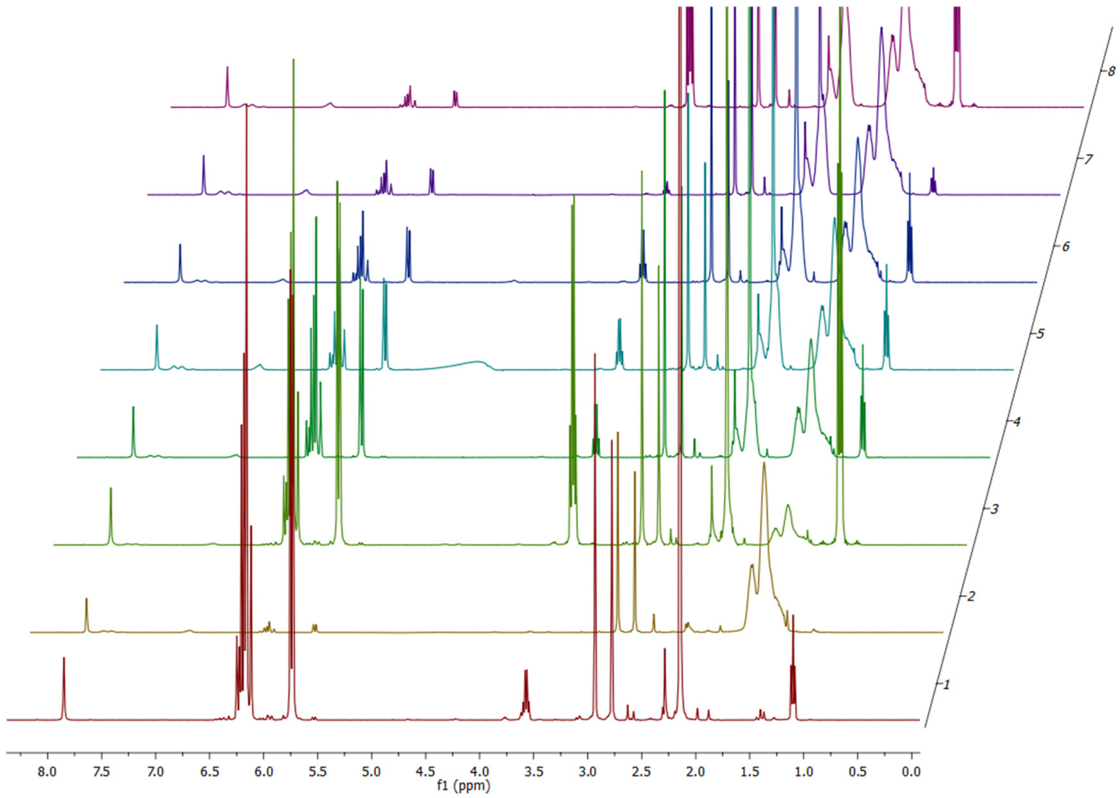

Figure S5. 400 MHz ^{1}H-NMR of reaction mixture samples recorded during the 500 mL galvanostatic *se*ATRP of AAm at $T = 10$ °C initiated by cellulose(H)-BriB.

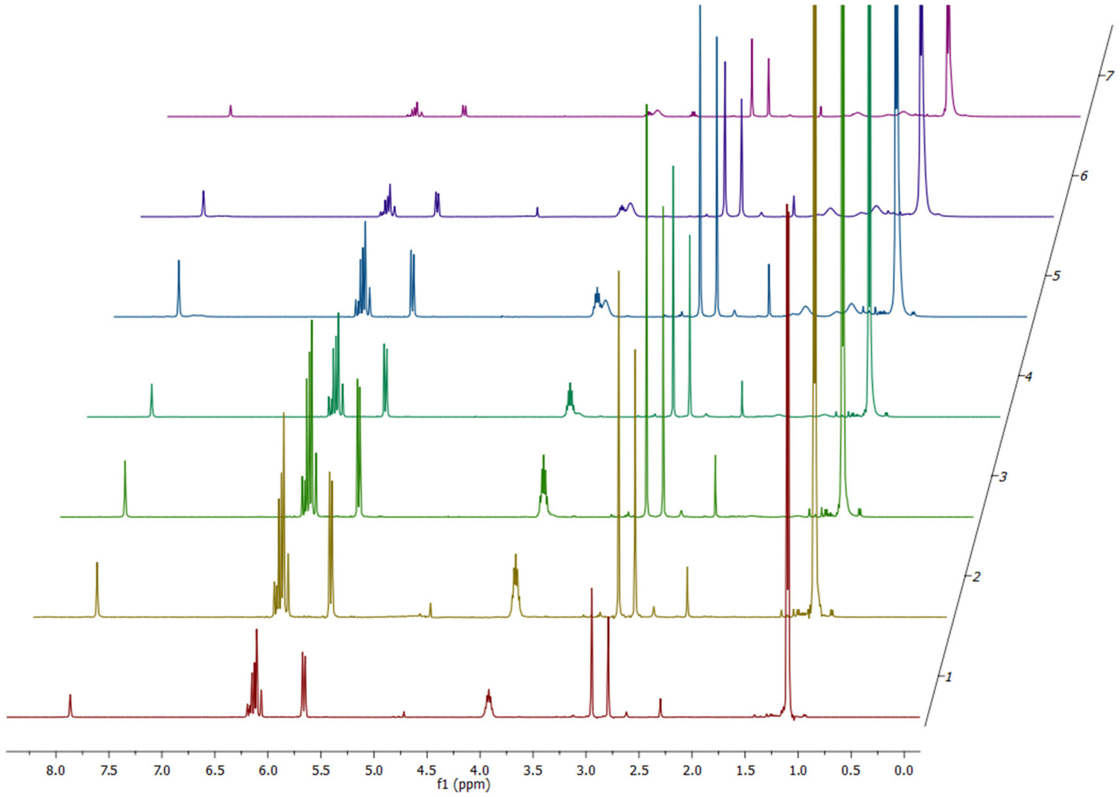

Figure S6. 400 MHz ^{1}H-NMR of reaction mixture samples recorded during the 500 mL galvanostatic *se*ATRP of NIPAAm at T = 10 °C initiated by cellulose(H)-BriB.

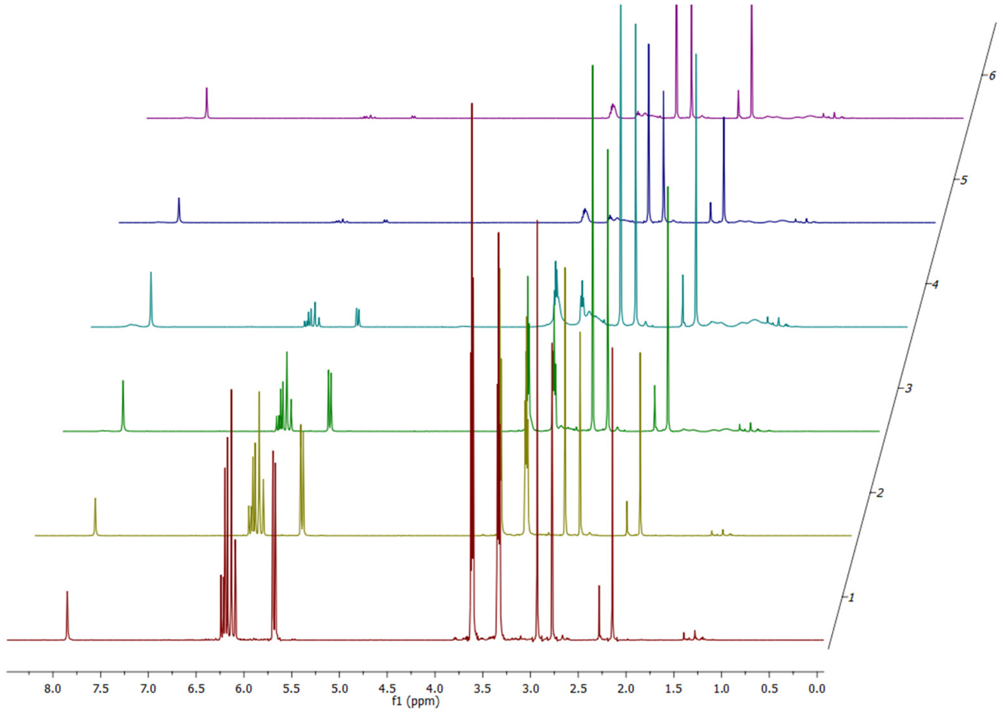

Figure S7. 400 MHz ^{1}H-NMR of reaction mixture samples recorded during the 500 mL *se*ATRP of HEAAm at T = 10 °C initiated by cellulose(H)-BriB.

S4. Additional details of ABIL quantification

To quantify the amount of [TMG][OAc] retained in the cellulose pulp-cellulose(H)-PNIPAAm film an extraction test is used. [1] Briefly, a film sample is extracted into deuterium oxide (D_2O) for 24 h, and the extracts is analyzed by ^{1}H-NMR, using a known amount of DMF as an internal standard. In this extraction test, the amount of retained [TMG][OAc] is calculated from a calibration curve that correlates the integrals of the formamidic hydrogen of DMF (~7.99 ppm) and the integrals of the methyl groups of TMG (~2.96 ppm). Thanks to procedural improvements during the preparation of the film, they have only a residual amount of [TMG][OAc] (< 1.5 wt.%). For that reason, ^{1}H-NMR was performed for extraction test for final resultant film. **Figure S8** shows the spectra obtained with the assignment of signals that were used to ABIL calculations.

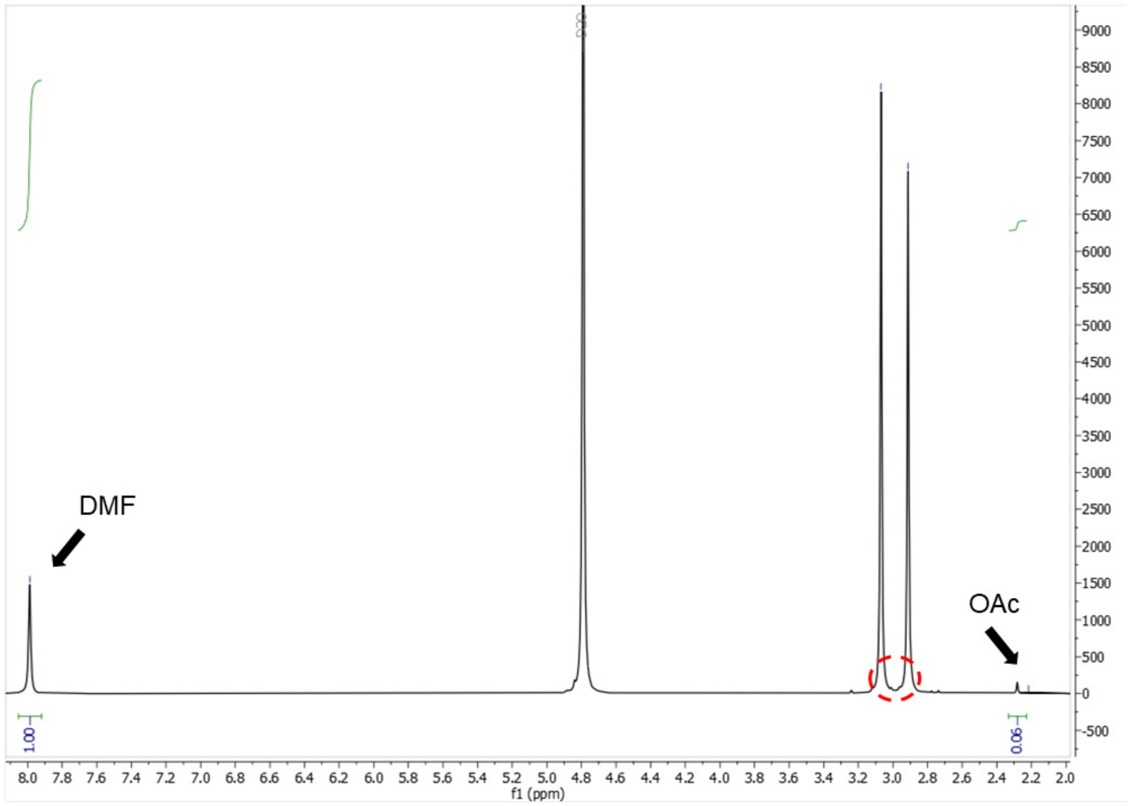

Figure S8. ^{1}H-NMR spectra of final film extracted in D_2O.

Normally, the TMG peak appears in the middle of the 2-CH_3 peaks of DMF (red circle). However, as can be seen, no peak appeared at this time. For such reason the adaptation of the quantification method was necessary. The peak of OAc was used this time for ABIL quantification.

S5. SEM images of cellulose-poly(*N*-alkyl)acrylamide-Br materials.

SEM images of cellulose and cellulose-*g*-poly(*N*-alkylacrylamides) were taken before and after the polymerization (**Figures S9-S11**).

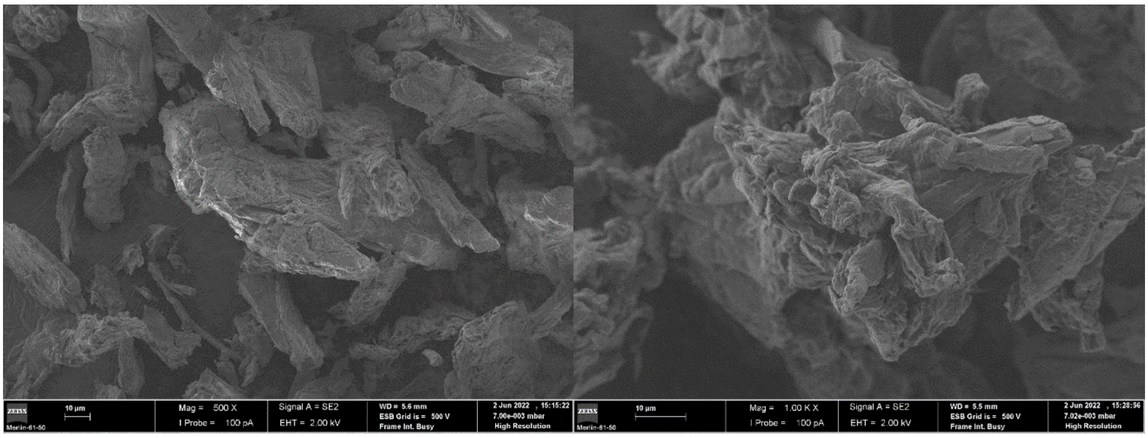

Figure S9. SEM images of a) Avicel PH-101 ® and b) cellulose(H)-BriB recorded at ×500 and ×1000 magnification respectively.

The most interesting change between cellulose and cellulose-*g*-poly(*N*-alkylacrylamides) is in the case of grafted PNIPAAm. Here two pore distributions appear. The first, larger, is of 10-20 μm size, with a second one of 0.5-1.0 μm. They appeared likely because of the separation of cellulose-*g*-PNIPAAm-Br from the polymerization mixture, exploiting the thermoresposive behavior of this material. Pores might have formed during the escape of water during the collapse of the polymeric structure when heated above the LCST.

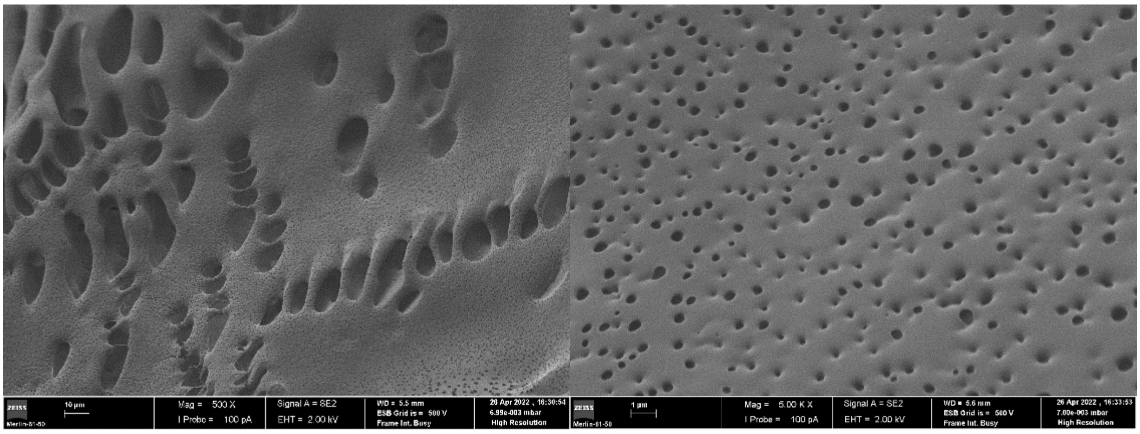

Figure S10. SEM images of cellulose(H)-*g*-PNIPAAm-Br recorded at ×1000 and ×5000 magnification respectively. Note the presence of two different populations of pore sizes of ~10-20 μm on a) and of 0.5-1 μm on. This fashion was only captured on cellulose(H)-*g*-PNIPAAm-Br.

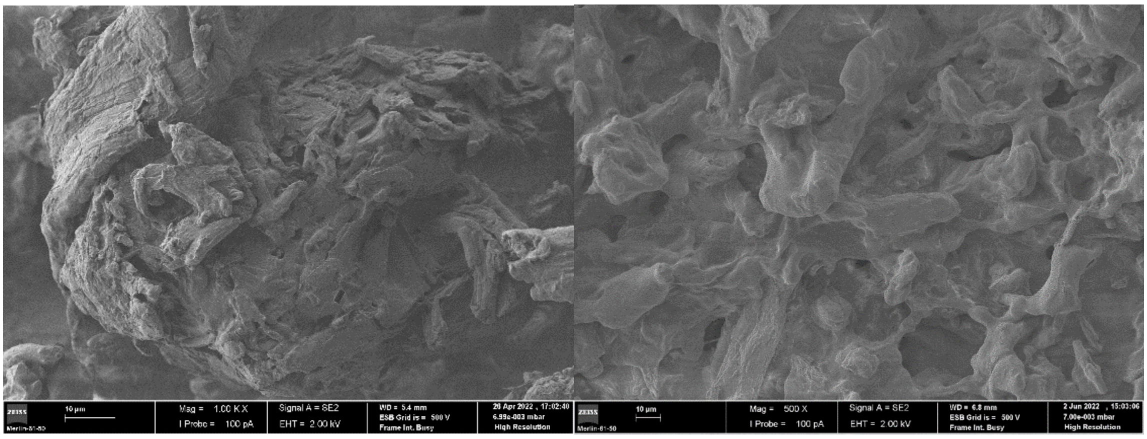

Figure S11. SEM images of cellulose(H)-*g*-PAAm-Br and cellulose(H)-*g*-PHEAAm-Br recorded at ×1000 and ×500 magnification respectively. Note the smoother surface of the material when PAAm and PHEAAm are grafted on the surface.

S6. Pictures and geometrical details of the reactors used in this work

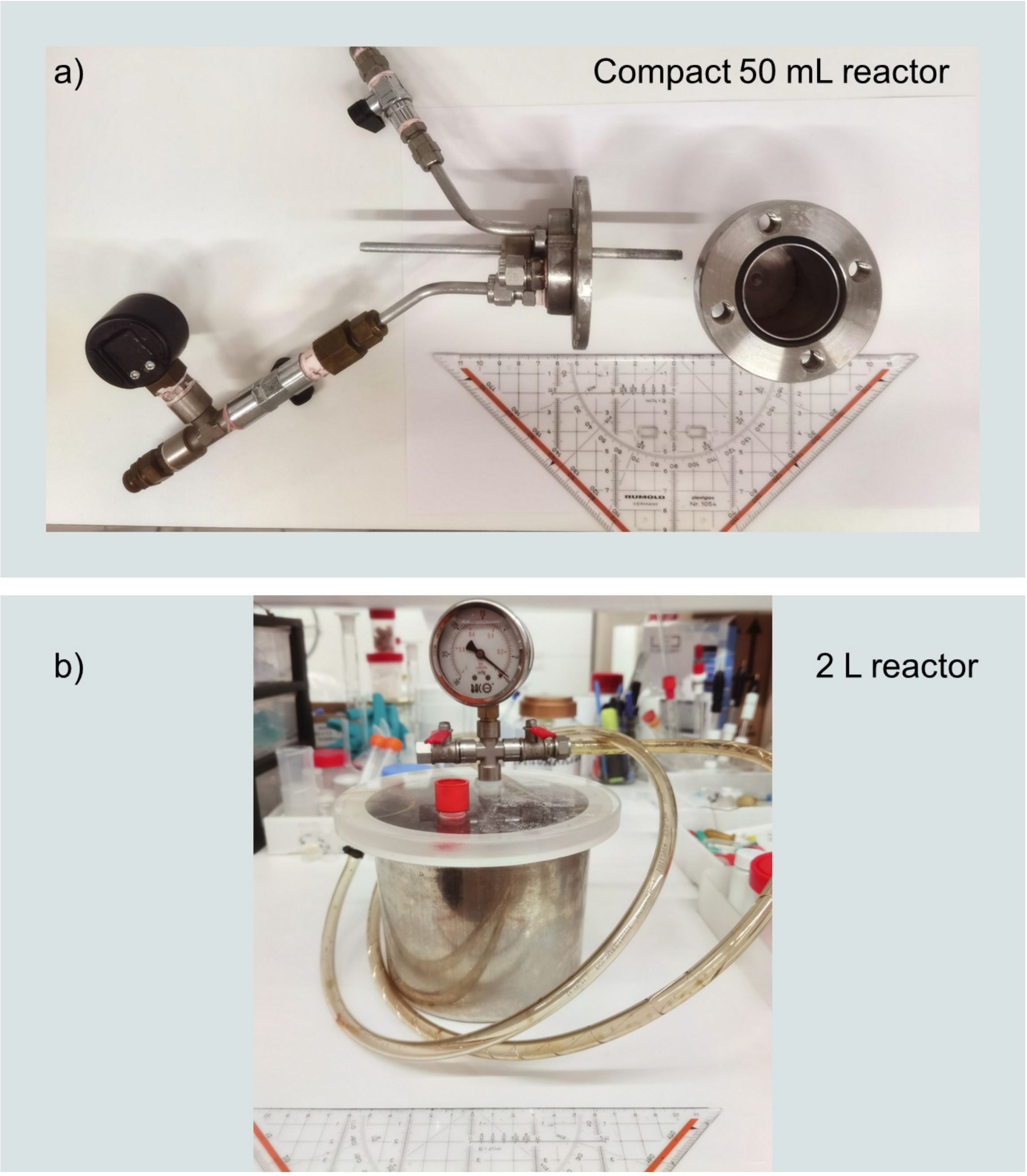

Figure S12. The SS304 electrochemical reactors used for *se*ATRP: a) 50 mL and b) 2.0 L reactor.

The electrochemical reactors used in this work have a volume of 50 mL or 2 L at full capacity and have been described in previous works. [2-4] These two reactors are composed by two fundamental parts: an SS304 body and a head. The head is equipped with a degassing tube, an open/close valve, a pressure gauge, a housing for a thermocouple (or other electronic conductor), an electrical connection for an aluminium CE, that serves as an anode, and a sampling point. Electrical insulation at the anode slot is provided by a

rubber insulator. The smallest reactor (**Figure S12a**) has an internal diameter of 4 cm, a height of 6.2 cm and a volume of 50 mL at full capacity. The wall thickness is 1 cm. The anode is an Al rod of 5 mm diameter that is immersed for 2.8 cm into the working solution when the volume is 40 mL. It is located 1 cm away from the centre of the reactor. The lid is 5 mm height. The biggest reactor (**Figure S12b**) has a diameter of 13.7 cm, a height of XXX cm and a volume of 2 L at full capacity. The wall thickness is approximately 2 mm. The anode used in this work is a 500 cm long aluminium coil that is immersed completely in the polymerization mixture, to have an approximate surface anodic area of 157.1 cm^2. The acrylic lid of this reactor can be damaged by aggressive solvents/monomers. Before the beginning of this work, it has been carefully sprayed with a water-repellent silicone coating to prevent damages, maintaining its long-term integrity. In the compact reactor, a 1.5 cm octahedral rare-earth magnet is used while an AlNiCo cross-shaped magnet is used in the 2 L reactor. The latter magnet mixes large volumes at low rpm (< 350-400 rpm) generating the necessary turbulence in solution for mass transfer (**Figure S13**).

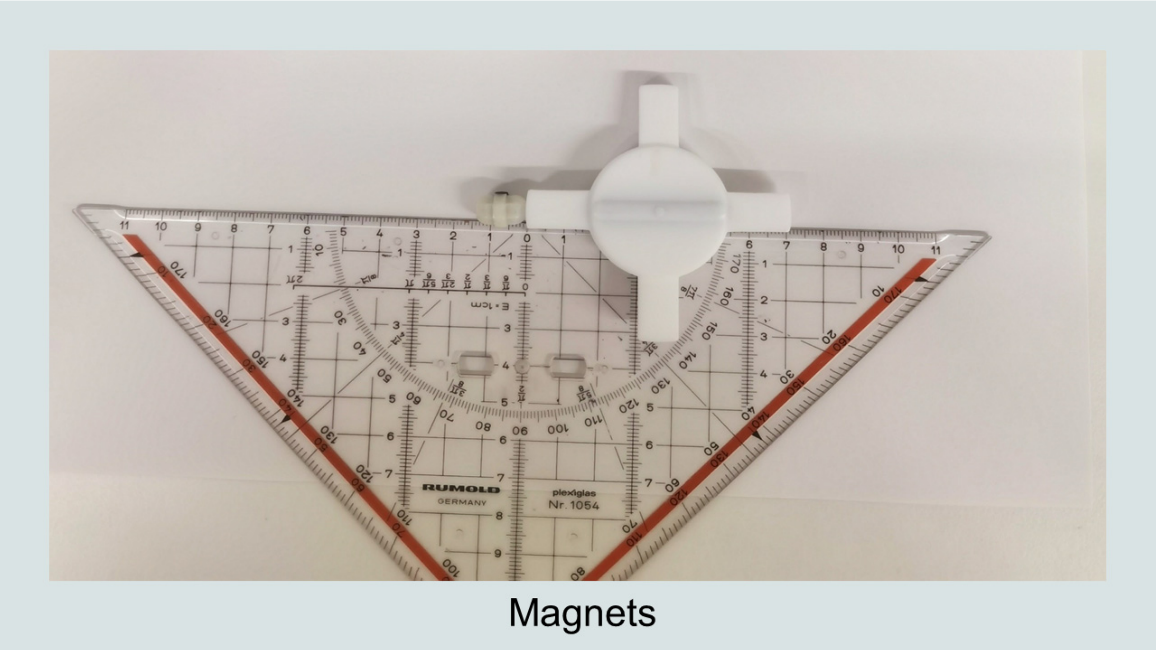

Figure S13. The magnets used in this work for efficient mixing.

Table S1 summarizes the geometrical characteristics of the reactors at different volumes according to the respective liquid height:

Table S1. Geometrical data of electrochemical reactors for polymerizations by galvanostatic *se*ATRP.

Entry	Volume (L)	Reactor type	Base diameter (cm)	Base area (cm^2)	Liquid height (cm)	Lateral area (cm^2)	Surface Area (S) (cm^2)	$\left(\frac{S}{V}\right)_n$ (cm^{-1}) [a]
**1**	0.04	50 mL	4.0	12.57	3.3	41.5	54.0	1.350
**2**	0.5	2.0 L	13.7	147.4	3.6	154.9	302.3	0.605
**3**	1	2.0 L	13.7	147.4	6.9	296.9	444.4	0.444

[a] On $\left(\frac{S}{V}\right)_n$ the subscript n is the entry number corresponding to the reaction, n = 1, 2, 3, 4, 5, 6.

Supporting References

1. Ribeiro, D.C.M.; Rebelo, R.C.; De Bon, F.; Coelho, J.F.J.; Serra, A.C. Process Development for Flexible Films of Industrial Cellulose Pulp Using Superbase Ionic Liquids. *Polymers (Basel)* **2021**, *13*, 1767, doi:10.3390/polym13111767.
2. De Bon, F.; Fonseca, R.G.; Lorandi, F.; Serra, A.C.; Isse, A.A.; Matyjaszewski, K.; Coelho, J.F.J. The scale-up of electrochemically mediated atom transfer radical polymerization without deoxygenation. *Chem. Eng. J.* **2022**, *445*, 136690, doi:10.1016/j.cej.2022.136690.
3. De Bon, F.; Ribeiro, D.C.M.; Abreu, C.M.R.; Rebelo, R.A.C.; Isse, A.A.; Serra, A.C.; Gennaro, A.; Matyjaszewski, K.; Coelho, J.F.J. Under pressure: electrochemically-mediated atom transfer radical polymerization of vinyl chloride. *Polymer Chemistry* **2020**, *11*, 6745-6762, doi:10.1039/d0py00995d.
4. De Bon, F.; Isse, A.A.; Gennaro, A. Towards scale-up of electrochemically-mediated atom transfer radical polymerization: Use of a stainless-steel reactor as both cathode and reaction vessel. *Electrochim. Acta* **2019**, *304*, 505-512, doi:10.1016/j.electacta.2019.03.032.
